# Supplementary material for: Evaluation of the performance and stability of early maturing orange-fleshed sweetpotato genotypes in selected areas in Ethiopia
Source: PLoS One. 2024 Oct 25;19(10):e0310273. doi: 10.1371/journal.pone.0310273 (PMC11508074; doi:10.1371/journal.pone.0310273)
Supplement: S1 Table — (DOC) [file pone.0310273.s002.doc]

Table 1. List of orange-fleshed sweetpotato genotypes used for the study

| No | Code | Genotype name | Source of genotypes | Root flesh color |
| --- | --- | --- | --- | --- |
| 1 | G1 | MUSG014052-51-5 | CIP-Udanda | Deep orange |
| 2 | G2 | MUSG014001-3-7 | CIP-Udanda | Intermediate Orange |
| 3 | G3 | 13NC9350A-9-3 | CIP-Udanda | Deep orange |
| 4 | G4 | CN1448-49-26-12 | CIP-Udanda | Deep orange |
| 5 | G5 | CN1448-49-28-9 | CIP-Udanda | Orange |
| 6 | G6 | 107031-18-5 | CIP-Udanda | Light Orange |
| 7 | G7 | 105413-5 | CIP-Udanda | Light orange |
| 8 | G8 | 105413-13 | CIP-Udanda | Intermediate Orange |
| 9 | G9 | CORDNER-15-2 | CIP-Udanda | Intermediate orange |
| 10 | G10 | Alamura (Check) | Ethiopia | Deep orange |

Source: National root crops research program, Hawassa Research Center, 2024.
