# Supplementary material for: Evaluation of the performance and stability of early maturing orange-fleshed sweetpotato genotypes in selected areas in Ethiopia
Source: PLoS One. 2024 Oct 25;19(10):e0310273. doi: 10.1371/journal.pone.0310273 (PMC11508074; doi:10.1371/journal.pone.0310273)
Supplement: Attachment — Submitted filename: Response to Reviewers letter.doc [file pone.0310273.s003.doc]

**A letter for responding to reviewers comments**

**Dear Dr. Kun Lu,**

Thank for giving us the opportunity to resubmit a revised version of our research paper titled “**Evaluation of the performance and stability of early maturing orange-fleshed sweetpotato genotypes in selected moisture stress areas in Ethiopia”** to *PLOSONE Journal.* We appreciate the time and effort that you and the reviewers have dedicated to providing your valuable feedbacks on our manuscript. We are grateful to the reviewers for their insightful comments on our paper. We have been able to incorporate changes to reflect most of the suggestions provided by the reviewers. Additionally, in order to properly reflect the content of the manuscript, the title was modified. The abstract, introduction, statistical models were explained in detail, and discussion including reference sections were thoroughly revised and also plosone formatting and style was followed as much as possible. Each change to the manuscript was marked with track changes.

The manuscript underwent a rigorous review with the assistance of two senior research scholars.

1. **Dr. Zerihun Yemataw** ([zerihunyemataw2@gmail.com](mailto:zerihunyemataw2@gmail.com)), Cell phone: +251911960755, senior plant breeder and geneticist, Sidama Agricultural Research Institute, Hawassa, Ethiopia

2. **Dr. Fekadu Gurmu** (fekadugb@gmail.com), Cell phone: +251911743625, senior plant breeder and geneticist, Ethiopian Institute of Agricultural Research, Addis Ababa, Ethiopia

Here is a point-by-point response to the reviewers’ comments and concerns.

**Comments to Authors from Editor**

1. **When submitting the amended version of paper, it was suggested to ensure that our manuscript meets PLOS ONE's style requirements, including those for file naming**.

**Response:** [Agreed to take action]. Thank you for pointing this out. As a result, we thoroughly reviewed the entire manuscript from the title to the supporting information that complies with PLOS ONE's style guideline. All changes made are indicated with track change in the document

2. **In your Methods section, please provide additional information regarding the permits you obtained for the work. Please ensure you have included the full name of the authority that approved the field site access and, if no permits were required, a brief statement explaining why.**

**Response**: No permits are needed; a brief explanation is provided in the main manuscript's materials and methods section.

3. **We suggest you thoroughly copyedit your manuscript for language usage, spelling, and grammar. If you do not know anyone who can help you do this, you may wish to consider employing a professional scientific editing service.**

**Response:** Thank you for pointing out this comment. We agree with this comment. Thus, we have invited two senior research scholars to critically review the manuscript in accordance with PLOS ONE guidelines including all technical issues like language, spelling, grammatical errors, and scientific soundness. Changes were made to all parts of manuscript that is indicated in track change.

**Comments to Authors from Reviewers**

**1**. **Is the manuscript technically sounds, and do the data support the conclusions?**

Reviewer #1: Yes

Reviewer #2: Yes

**Response:** [Accepted]. Thank you for pointing this out. We agree with this comment. As a result, we thoroughly reviewed the entire manuscript from the title to the supporting information. All changes made are indicated with track change in the document

**2**. **Has the statistical analysis been performed appropriately and rigorously?**

**Reviewer #1: No**

**Reviewer #2: Yes**

**Response: [Yes].** Thank you for this suggestion. It would have been interesting to investigate this further. However, in case of our study, we used many forms of analysis to achieve our objective. For instance, we computed ANOVA, GXE and stability using step-wise analysis results.

**3. Have the authors made all data underlying the findings in their manuscript fully available?**

**Reviewer #1: Yes**

**Reviewer #2: No**

**Response: [**data are available in manuscript and in supporting information**].** Nearly all of the data are included in the manuscript. The remainder was provided as supporting information.

**4. Is the manuscript presented in an intelligible fashion and written in Standard English?**

**Reviewer #1: Yes**

**Reviewer #2: No**

**Response:** [Corrective action is agreed upon]. We have thus rewritten our paper and made a lot of amendments on the titles, abstract body, materials and methods, statistical models, result and discussion, conclusion references section, etc. for the sake of scientific standards and to fulfill the *PLOS ONE* standard.

With best regards,

Bililign Mekonnen

Senior researcher and coordinator of national root crops research program in Ethiopia, Hawassa, Ethiopia
