# Supplementary material for: Evaluation of the performance and stability of early maturing orange-fleshed sweetpotato genotypes in selected areas in Ethiopia
Source: PLoS One. 2024 Oct 25;19(10):e0310273. doi: 10.1371/journal.pone.0310273 (PMC11508074; doi:10.1371/journal.pone.0310273)
Supplement: Attachment — Submitted filename: Manuscript_Reviewed.doc [file pone.0310273.s004.doc]

Evaluation of the performance and stability of early maturing orange-fleshed sweetpotato genotypes in selected moisture stress areas in Ethiopia

**Bililign Mekonnen^1*^, Fekadu Gurmu^2^, Damtew Abewoy^3^, Kanko Chuntale^4^, Birhanu Habete^5^**

**1** Sidama Region Agricultural Research Institute (SIRARI), Hawassa Agricultural Research Centre, Hawassa, Ethiopia, **2** Ethiopian Institute of Agricultural Research (EIAR), Addis Ababa, Ethiopia, **3** Ethiopian Institute of Agricultural Research (EIAR), Wondo Genet Agricultural Research Center, WondoGenet, Ethiopia, **4** South Ethiopia Agricultural Research Institute (SEARI), Arba Minch Agricultural Research Center, Arba Minch, Ethiopia, **5** Ethiopian Institute of Agricultural Research (EIAR), Fogera National Rice Research and Training Center, Fogera, Ethiopia

* bililign.m@gmail.com

# Abstract

Orange-fleshed sweetpotato (OFSP) varieties offer prospects for food and nutrition security in the face of climate change, arable land scarcity, and population growth as they can produce higher yields on small plots of land in a short period of time. To address these problems, it is vital to develop early-maturing varieties that give better root yield within the narrow crop production window. The study aimed to identify early-maturing and high-yielding genotypes. The field experiment was conducted using ten genotypes in a randomized complete block design with three replications across three locations over two years (2021–2022). Data were collected on root yield and yield-related traits, sweetpotato virus disease reactions (SPVD), root dry matter (DMC), and beta-carotene contents (BCC). A combined analysis of variance and genotype plus genotype by environment interaction (GGE) bi-plot were used to analyze the performance and stability of the genotypes. The significant differences (p<0.001) were observed across locations and among test genotypes, signifying the existence of differences among genotypes and the test locations. The results of the combined analysis showed that genotype G3 (13NC9350A-9-3) outperformed the check in all environments, with a yield advantage of 41.4 t ha^-1^ over the check variety. The GGE biplot also revealed that G3, located at the vertices of the polygon, indicated that it performed the best. It also showed consistent performance across locations, a good level of resistance to SPVD with a score of 1.39 on a scale of 1 to 9, acceptable level of DMC (30.0%) and BCC (5.5 mg/100 g), matured in three months, and flowering profoundly (for future crossing purposes). Overall, G3 has the potential to significantly contribute to efforts to relieve hunger and food insecurity caused by climate change in Ethiopia. Therefore, it can be recommended for verification and release for wider production in the country.

**Introduction**

Sweetpotato is an important crop that can best adapt and thrive under low moisture stress areas. It can also give better yield than other crops under low input conditions. The orange-fleshed sweetpotato group has added advantage of the white sweetpotato group to supplying a healthy and nutrition food for areas where food and nutritional security are critical problems [1-3].

Among the five critical micro-nutrient mineral deficiency nutrients, vitamin A has remained a serious health concern in resource-poor rural areas around the world [4, 5]. Various strategic approaches, viz, supplementation, fortification, diversification, and biofortification, are being recommended as possible avenues to curb malnutrition. Cultivation and consumption of biofortified crops commonly grown in an area is thought to be one of the most affordable strategies to addressing malnutrition-related issues. To this effect, biofortified orange-fleshed sweetpotato cultivars have significant role to combat vitamin A deficiency [6, 7]

Following the global climate change driven moisture stress caused by prolonged drought is currently threatening sustainable crop production and posing existential food problems to smallholder farmers [3]. This problem is currently affecting sweetpotato production and productivity in Ethiopia. Some sweetpotato varieties that have drought or moisture stress tolerant quality are of better resort to this challenge. However, the response of these varieties depends on factors like the onset and severity of drought [8]

One of the mechanisms to overcome the effect of drought is drought escape through early bulking and maturity [9]. Sweetpotato cultivars in Ethiopia are classified into three groups according to maturity: early maturing within 3 months, medium maturing 4 to 5 months, and late maturing > 5 months [7].

Genetic material from 16 polycross families was introduced as botanical seeds from CIP- Uganda in 2016 to conduct the current study. More than 420 seeds were characterized based on different traits, and only the best ones were advanced to the next step and evaluated in several breeding stages at different locations. As a result, this national variety trial was conducted with ten genotypes (nine selected including one check) at three locations (Hawassa, Koka, and Arba Minch) over two consecutive years (2021-2022) with the objective of identifying early-maturing varieties that can mature early and escape the effects of low moisture stress caused by prolonged periods of drought.

**Materials and methods**

##  Description of study areas and its weather conditions

The trial was conducted for two consecutive years (2021 and 2022) at three locations: Sidama (Hawassa), South (Arba Minch) and Oromia (Koka) regions in Ethiopia. Concerning the weather conditions during sweetpotato growing seasons, in 2021, Arba Minch, Hawassa and Koka areas received 541.2, 993.5 and 523.6 mm rain, respectively during the growing season of sweetpotato. However, the growing season rainfall was only 291.2, 601 and 470 mm in 2022, in that respective order. This showed that sweetpotato plants received lower amount of rainfall in 2022 than 2021; and the rainfall received during the growing season was the lowest at Arba Minch, intermediate at Koka and the highest at Hawassa (S1 Data). Thus, sweetpotato plants suffered moisture stress for three months of the growing period in 2022 as rainfall met evapotarnspiration requirement only in October and September in Arba Minch and Koka areas, respectively, which coincided with periods of root bulking and establishment in respective order marking greater loss in Koka area than Arba Minch area. Moreover, rainfall during August and September was always below the crop requirement in Arba Minch whether the year is good or bad as was October and November in Koka. In Hawassa, August and September were occasionally drier [S1 Data].

**Testing sites permission**

This work is a part of the Ethiopian Institute of Agricultural Research (EIAR). The institute already had linkages with regional agricultural research centers and agricultural universities in Ethiopia to collaborate on any research activities. Furthermore, when designing field experiments, any field research activities must go through a review process that includes testing locations. The activity can be carried out at the designated locations (sites) without any limitations or requests for site authorization once it has successfully completed this process.

**Experimental materials**

Genetic materials derived from 16 polycross families were introduced from Uganda in the form of botanical seeds in 2016. To establish the field trial, over 420 botanical seeds were characterized based on various traits, and selected genotypes were tested at various breeding stages. Then, 10 genotypes (nine selected and one check) were tested in the national variety trial at three locations (Hawassa, Koka, and Arba Minch) over the course of two years (2021 to 2022), as shown in [S1 Table].

**Experimental design and field management**

The field experiment was conducted using a Randomized Complete Block Design (RCBD) with three replications. Each genotype is planted on a plot size of 2.4 m wide and 3 m length (a total plot area of 7.2 m^2^). Each plot consisted of four rows/ridges each raw containing 10 plants/row and a total of 40 plants/cuttings/plot. The spacing between plants and rows were 0.3 m and 0.6 m, respectively. The spacing used between replications was 1.5 m. In each plot, one plant (cutting) of 30 cm length with 5-8 nodes was planted in the prepared holes by burying one-third of the plant in the soil. After one week, dead vines (plants) were replaced by new ones (to maintain uniformity of plant population per plots), and after the fourth week, earthening up was done, and all plots were manually weeded as per the recommendation for sweetpotato production in Ethiopia [10]

**Data collection procedure**

All agronomic data were collected from the two central rows, i.e., excluding the two plants grown at both ends of the row and the two border rows. Data on diseases severity was collected before one month of harvesting and data on root- yield, and yield-related traits were recorded at harvest.

Sweetpotato Virus Disease (SPVD): - recorded on a plot basis, on a scale of 1 to 9, 1 indicating no virus symptoms, 2 indicating unclear virus symptoms; 3 = Clear virus symptoms for < 5% of plants per plot; 4 = Clear virus symptoms for 6–15% of plants per plot; 5 = Clear virus symptoms for 16–33% of plants per plot; 6 = Clear virus symptoms for 34–66% of plants per plot (i.e. > 1/3 and <2/3); 7 = Clear virus symptoms for 67–99% of plants per plot (2/3 to almost all); 8 = clear virus symptoms for all plants per plot ( not stunted); and 9 = Severe virus symptoms for all plants per plot (stunted) as per the procedure suggested by [11]. Aboveground fresh weight biomass (t ha^-1^): The weight of above ground parts of the two middle rows was obtained and converted to t/ha. Harvest index (HI): determined as a ratio of economical yield (fresh root weight) to total weight (above ground fresh weight + fresh root weight) on fresh weight basis. Root length (cm): the length of a storage root that measured from distal to proximal on five randomly selected plants during harvest. Root girth (cm): the diameter of the storage root measured from the middle portion of the root. The girth of all the storage roots of each of the sampled plants were measured with a caliper, divided by number of storage roots from all plants sampled and expressed in centimeter. Root yield (t ha^-1^): is the weight of clean, uninfected storage roots weighing 100g-500g. It was calculated by weighing all of the storage roots harvested from the harvestable plot with a beam balance and expressing the results as ton/ha.

**Statistical analysis**

**Analysis of variance**

Prior to performing the combined analysis, a simple ANOVA was computed for each location (not shown here), and then the homogeneity of error variance across locations over years was checked using the F-ratio, which was found to be non-significant, and then the combined analysis was performed accordingly. Finally, a combined analysis of variance was performed using SAS software version 9.3 (SAS Institute Inc., 2003). The Fisher's protected least significant difference (LSD) test was used to compare the treatments at 1% and 5% probability levels as procedure suggested by [12]

The following statistical model was used for combined analysis of variance over locations:

$Y_{\text{ijkl}}=\mu+G_{i}+L_{j}+Yk+\text{GL}_{\text{ij}}+\text{GY}\text{ik}+\text{LY}\text{jk}+\text{GLY}\text{ijk}+B_{l(\text{jk})}+\varepsilon_{\text{ijkl}}$

Where: Y_ijkl_ is observed value of genotype *i* in block *l* nested in (location *j* and year *k)*, μ is grand mean, G_i_ is genotype effect, L_j_ is location effect, Y_k_ is year effect, GL_ij_, GY_ik_ and GLY_ijk_ are the interaction effect of genotype *i* with location *j*, genotype *i* with year k and genotype *i* with location *l* and year *k*, є_ijkl_ is error (residual) effect.

**Genotype by environment interaction (GEI) analysis**

The data were analyzed in GenStat (18^th^ version) for GEI and stability of genotypes using the GGE biplot procedure [13-15]

The model for the GGE biplot based on singular value decomposition (SVD) of the principal components is:

$$\bar{Y}_{\text{ij}}-\mu_{i}-\beta_{j}=\sum\lambda_{k}\alpha_{\text{ik}}\gamma_{\text{jk}}+\varepsilon_{\text{ij}}$$

Where: $\bar{Y}_{\text{ij}}$is the performance of genotype *i* in environment *j*, $\mu$ is the grand mean, $\beta_{j}$ is the main effect of environment *j*, *k* is the number of principal components (PC); $\lambda_{k}$is singular value of the *k*^th^ PC; and $\alpha_{\text{ik}}$and$\gamma_{\text{jk}}$are the scores of *i*^th^ genotype and *j*^th^environment, respectively for PC_k_; $\varepsilon_{\text{ij}}$ is the residual associated with genotype *i* in environment *j*.

**Results and discussion**

**Analysis of variance of root yield and yield-related traits**

Results of combined analysis of variance revealed the presence of a highly significant (p<0.001) difference between genotypes for all the traits considered (Table 1). There was also a significant (p<0.001) effect of location by genotype interaction for all characteristics except SPVD, the observed difference indicating that genotypes respond differently across locations. The presence of significant genotype by locations interaction complicates the process of variety recommendation to wider areas [16], hence demanding further stability analysis to identify a stable genotype across locations as is essential suggested by [17]. The three-way interaction effect (Genotype x Location x Year) was significant for all traits, showing that these significant differences might be attributed to genotypic differences, growing season, cultural management, and environmental conditions. The non-significant differences were observed for SPVD across locations; this may imply that the absence of significant variation in SPVD was either due to the absence of a suitable environment for the virus to appear or an absence of vectors or carriers that spread the virus from plant to plant by sucking insects near research sites [18].

Table 1. Analysis of variance for agronomic traits of 10 OFSP genotypes evaluated across three locations over two years

| Source of variance | df | Mean squares | | | | | |
| --- | --- | --- | --- | --- | --- | --- | --- |
|  |  | SPVD  (1-9) | Root length  (cm) | Root girth  (cm) | Above ground biomass  (t ha-1) | Root yield  (t ha-1) | Dry matter content  (%) |
| Rep (LxY) | 10 | 0.33ns | 67.15*** | 16.00** | 743.50*** | 6.17ns | 0.0002ns |
| Genotype (G) | 9 | 3.37*** | 126.47*** | 59.65*** | 592.64*** | 1807.89*** | 0.09*** |
| Location (L) | 2 | 1.59ns | 1135.15*** | 2893.19*** | 25839.80*** | 1632.88*** | 0.06*** |
| Year (Y) | 1 | 10.58*** | 50.47** | 61.34*** | 870.58** | 13.68ns | 0.0003ns |
| GxL | 18 | 3.43*** | 28.86*** | 12.94** | 1075.73*** | 269.75*** | 0.06*** |
| GxY | 9 | 2.30** | 3.47ns | 15.02** | 152.00ns | 31.95*** | 0.0002ns |
| LxY | 2 | 17.35*** | 12.62ns | 19.51** | 15925.83*** | 462.35*** | 0.08*** |
| GxLxY | 18 | 1.57** | 5.93ns | 15.31** | 142.64ns | 76.95*** | 0.0002ns |
| Error | 108 | 0.73 | 5.41 | 6.00 | 175.87 | 5.17 | 0.0002 |

Where, 1-9 scale (1 = immune, 9 =Susceptible, hence 1-3 = resistant, 4 – 6 = medium and 7-9 = susceptible)

**Performance of genotypes for mean root yield across location and years**

A highly significant variation was observed for root yield between genotypes across three locations over years [Table 2]. The presence of differential response of genotypes to these variations might be attributed to the genetic differences of genotypes [7]. The highest root yield of 49 ton/ha produced by genotype G3 (13NC9350A-9-3) from Arba Minch in 2021 and 42.5 t/ha from Koka site in 2022 with overall mean root yield of 36.2 t/ha, which is 41.4 % yield advantage over the standard check variety. Although the performance of some genotypes was very good at Koka and Arba Minch locations, their performance was poor at Hawassa locations in both years except for one genotype. The genotype G3 gave high root yield in all environments for root yield, whether the year was good or bad [Table 2]. The five genotypes G4 (CN1448-49-26-12), G6 (107031-18-5), G7 (105413-5), G8 (105413-13), and G9 (CORDNER-15-2) produced the lowest root yields (less than 10 t/ha), indicating their poor performance (very low yielders) under the evaluated environmental conditions. When selecting sweetpotato genotypes early maturation and high root yield is one of among an important trait to be considered [19].

Table 2. Root yield (t/ha) performance of OFSP genotypes evaluated across locations and over years

| Code | Genotype name | **2021** | | | | **2022** | | | | **Genotype by Environment** | Yield advantage over standard check (%) |
| --- | --- | --- | --- | --- | --- | --- | --- | --- | --- | --- | --- |
|  |  | **Hawassa** | **Koka** | **ArbaMinch** | **Mean** | **Hawassa** | **Koka** | **ArbaMinch** | **Mean** |  |  |
| G1 | MUSG014052-51-5 | 14.0 | 13.5 | 48.9 | 25.5 | 13.7 | 8.7 | 32.6 | 18.3 | 21.9 | -14.5 |
| G2 | MUSG014001-3-7 | 10.7 | 15.9 | 30.6 | 19.0 | 12.9 | 17.4 | 20.4 | 16.9 | 18.0 | -29.7 |
| G3 | 13NC9350A-9-3 | 33.7 | 28.5 | 49.0 | 37.0 | 30.3 | 42.5 | 33.6 | 35.5 | 36.2 | 41.4 |
| G4 | CN1448-49-26-12 | 7.0 | 12.5 | 8.0 | 9.2 | 6.9 | 20.2 | 7.3 | 11.5 | 10.3 | -59.8 |
| G5 | CN1448-49-28-9 | 11.6 | 26.4 | 32.3 | 23.5 | 15.3 | 30.0 | 28.3 | 24.6 | 24.0 | -6.3 |
| G6 | 107031-18-5 | 7.7 | 8.5 | 4.7 | 7.0 | 8.1 | 6.5 | 7.4 | 7.3 | 7.1 | -72.3 |
| G7 | 105413-5 | 9.9 | 22.2 | 41.1 | 24.4 | 16.2 | 31.2 | 27.4 | 24.9 | 24.7 | -3.5 |
| G8 | 105413-13 | 6.5 | 10.0 | 4.2 | 6.9 | 7.2 | 7.7 | 7.0 | 7.3 | 7.1 | -72.3 |
| G9 | CORDNER-15-2 | 6.4 | 9.60 | 3.1 | 6.4 | 6.1 | 7.8 | 7.0 | 7.0 | 6.7 | -73.8 |
| G10 | Alamura (Check) | 13.2 | 23.4 | 41.3 | 25.9 | 14.8 | 36.3 | 24.6 | 25.2 | 25.6 | - |
| Mean | | 12.0 | 17.1 | 26.3 | 18.8 | 13.1 | 21.0 | 19.6 | 17.7 | 18.3 |  |
| LSD (5%) | | 3.7 | 4.2 | 4.8 | 4.2 | 3.4 | 3.9 | 3.5 | 3.6 | 3.9 |  |
| CV (%) | | 18.3 | 14.6 | 11 | 14.6 | 15.5 | 11.1 | 10.6 | 12.4 | 13.5 |  |

**Mean performance of genotypes for SPVD reaction, root length, root girth and above-ground biomass**

The combined analysis of variance revealed the presence of a significant difference (p<0.05) among	genotypes in response to SPVD. The severity of symptoms were assessed based on visual scoring and the obtained results were	ranged from 1.39 to 2.78, on a 1-9 rating scales, with a mean of 1.94 (Table 3). The current result showed that almost all of the genotypes tested demonstrated a high level of resistance or/ tolerance to SPVD with low score of < 3.0, which is fell between resistant ranges to SPVD severity [11, 18].

The absence of high severity levels among tested genotypes for SPVD may indicate the lack of a suitable environment for the virus to emerge, as well as the absence of vectors or carriers that spread the virus from plant to plant via sucking insects near research sites [20, 21]. The authors also explained that if disease-transmitting insects had not been present in the area, the disease would not have appeared or spread. While selecting and advancing sweetpotato genotypes for later stages, it is suggested to assess their sensitivity to SPVD reactions along with other desirable traits [19, 21]. There were significant differences (p<0.05) in root length across locations and years [Table 4]. The root length varied from 5.39 cm to 10.41 cm for genotypes G8 (105413-13) and G7 (105413-5), respectively, with an overall mean of 7.96 cm. In sweetpotato, root sizes (length and girth) are important traits to consider when selecting genotypes for earliness (early harvesting i.e. it matures in three month) because the size of the roots are characterized as lengthy roots (root length with more than 15 cm) and a small size (root girth/diameter with less that 3 cm) will be undesirable on the market. Furthermore, some genotypes with short roots of extremely big or small sizes fall within unmarketable root ranges and will be unappealing to the majority of consumers. Thus, it is critical to evaluate genotypes that produce both medium-sized along with medium root lengths during the selection process, as these categories fall into market classes (saleable).

Significant variations were observed among genotypes at three locations over years for aboveground biomass yield (t/ha). The maximum mean yield of 35.5 t/ha was recorded for G5 (CN1448-49-28-9) whereas the minimum mean yield of 25.2 t/ha obtained from check variety G10 (Alamura). The above-ground biomass of sweetpotato genotypes is utilized as livestock feed, which is one of the crop’s key trait and what makes it so popular in agricultural communities under land constrain [22]. Genotypes that have both a high root yield and a high above-ground biomass can be used for dual purposes (human consumption and feed for livestock). Gardiner [23] found that the harvest index signified the distribution of assimilation between economic and total plant biomass. The harvest index (HI) performance of genotypes revealed significant variation. The genotype G3 (13NC9350A-9-3) had the highest mean value of 61% of HI, while the genotype G9 (CORDNER-15-2) had the lowest mean value of 20% of HI. A genotype with a high harvest index value is believed to have a high efficiency in storage root production in relationship to its biological yield. The similar findings by [23] indicated that high yielding varieties have a higher harvest index than low yielding varieties in terms of fresh root yield

Table 3. Mean performance of 10 OFSP genotypes for various traits across locaction over years

| Code | Genotypes | SPVD  (1-9 scale) | RL  (cm) | RG  (cm) | AGFW  (t ha^-1^) | HI  (%) |
| --- | --- | --- | --- | --- | --- | --- |
| G1 | MUSG014052-51-5 | 1.83 | 11.84 | 8.67 | 37.19 | 43.00 |
| G2 | MUSG014001-3-7 | 1.61 | 13.67 | 7.43 | 32.80 | 43.00 |
| G3 | 13NC9350A-9-3 | 1.39 | 15.10 | 9.03 | 27.74 | 61.00 |
| G4 | CN1448-49-26-12 | 1.72 | 10.51 | 6.47 | 24.50 | 32.00 |
| G5 | CN1448-49-28-9 | 2.78 | 13.76 | 9.87 | 41.24 | 49.00 |
| G6 | 107031-18-5 | 2.11 | 9.71 | 5.98 | 30.73 | 25.00 |
| G7 | 105413-5 | 2.55 | 12.06 | 10.41 | 34.31 | 50.00 |
| G8 | 105413-13 | 1.78 | 7.98 | 5.39 | 30.61 | 21.00 |
| G9 | CORDNER-15-2 | 1.66 | 8.09 | 6.51 | 26.47 | 20.00 |
| G10 | Alamura (Check) | 1.99 | 15.06 | 9.80 | 22.90 | 55.00 |
| Mean | | 1.94 | 11.78 | 7.96 | 30.85 | 40.00 |
| LSD (0.05) | | 0.78 | 3.34 | 4.32 | 8.67 | 9.00 |
| CV (%) | | 26.30 | 23.40 | 27.00 | 28.80 | 24.10 |

Where, 1-9 scale (1 = immune, 9 =Susceptible, hence 1-3 = resistant, 4 – 6 = medium and 7-9 = susceptible); SPVD=Sweetpotato virus disease, RL=Root length, RG=Root girth, AGFW=above ground biomass, HI=Harvest index

**Performance of OFSP genotypes for root dry matter, beta-carotene contents and flowering potential**

Dry matter content particularly in orange-fleshed sweetpotato varieties is of paramount and highly desired eating quality trait by most African farmers including Ethiopians [7]. In this study, the tested genotypes showed significant difference across locations over years for DMC (%) that was ranged from 24.0% to 31%, with overall mean of 27.0% (Table 4). The highest mean of 31% DMC recorded for check variety (Alamura), while the lowest mean of 24.0% obtained from genotype G6 (107031-18-5). Three genotypes such as G3 (13NC9350A-9-3), G7 (105413-5) and check variety Alamura had DMC of 30%, 30%, and 31%, respectively. However, no genotype exceeded the check variety (Alamura) as compared to it (Table 4). Beta-carotene (BCC) which is a precursor to vitamin A is also one of the most important components of OFSP. The tested genotypes for BCC differed significantly (p<0.05) among locations over years as estimated based on root flesh color chart developed by [24]. According to [24] color chart standards, the deeper orange root flesh color of OFSP genotype indicates a higher beta-carotene concentration. Accordingly, check genotype (Alamura) had the highest mean value of 7.46 gram/100g, whereas genotype G9 (CORDNER-15-2) had the lowest mean value of 3.48 gram/100g. During OFSP genotype selection, most favorable genotypes may be defined as orange/intermediate orange ranging from 5.08 to 8.36 gm/100g on a fresh weight basis using standard procedure developed by [24]. The candidate variety's BCC value is within the acceptable range, i.e. 5.5 gram/100g on a fresh weight basis. A genotype with a relatively high beta-carotene content combined with a higher root yield is required to select a suitable genotype for production as a systematic diet in areas where there is a nutritional crisis [25]. In terms of flowering ability, most of the evaluated genotypes seldom produced flowers under test environments; however, genotypes with higher flowering potential are desired since they may be used as parents for future breeding purposes. As a result, genotype G3 performed very well for this trait and can be used as a parent for future crossing purposes upon further study of the traits of interest (Table 4).

Table 4. Mean dry matter content, flesh colour and flowering ability of the evaluated sweetpotato genotypes

| Code | Genotypes | DMC  (%) | BCC*  (gm/100g) | Flowering ability of evaluated genotypes |
| --- | --- | --- | --- | --- |
| G1 | MUSG014052-51-5 | 26.00 | 7.11 | Mostly not flowering or 2% |
| G2 | MUSG014001-3-7 | 25.00 | 3.65 | Mostly not flowering or 3% |
| G3 | 13NC9350A-9-3 | 30.00 | 5.50 | 95% flowering |
| G4 | CN1448-49-26-12 | 27.00 | 3.68 | Mostly not flowering or 3% |
| G5 | CN1448-49-28-9 | 27.00 | 3.67 | Mostly not flowering or 5% |
| G6 | 107031-18-5 | 24.00 | 3.72 | Mostly not flowering or 3% |
| G7 | 105413-5 | 30.00 | 3.75 | Mostly not flowering or 5% |
| G8 | 105413-13 | 25.00 | 3.50 | Mostly not flowering or 3% |
| G9 | CORDNER-15-2 | 25.00 | 3.48 | Mostly not flowering or 3% |
| G10 | Alamura (Check) | 31.00 | 7.46 | Mostly not flowering or 10% |
| Mean | | 27.00 | 4.60 |  |
| LSD (0.05) | | 2.00 | 0.55 |  |
| CV (%) | | 15.50 | 7.20 |  |

Where, DMC= Dry matter content, BCC=Beta-carotene content= estimated on fresh weight basis based on based on colour chart

**Genotype by Environment and Stability of Genotypes based on GGE Biplot Analysis**

Analysis of GGE biplot showed the relative performance of 10 OFSP genotypes for root yields across locations as shown in (Fig 1). The first two principal components (PC1 & PC2) explained 84.14% and 10.88% with 95.02% overall observed variation, respectively, entailing that they adequately explained the GGE (Fig 1). Regarding an interpretation of values from GGE biplot analysis, a large and positive PC scores for a specific genotype indicate a higher average value, while those with large negative PC scores imply a lower value [13, 15]. Accordingly, genotypes G3, G10, G7, G1 and G5 produced relatively the highest root yields. Conversely, G6, G8, G9 and G4 produced the lowest mean root yields. Genotypes G5, G2 and G7 had low PC2 scores that are close to zero, indicating that they were relatively stable. Among which Genotype G7 is more stable but less yielder as compared to G3 and G10 (Fig 1). The vertex genotypes at the polygon G1, G3 G4, G9 and G8 performed either best or poorly, agreeing with the previous reports on evaluations of sweetpotato genotypes based on a GGE biplot analysis [21, 26]. Genotype G3 performed well almost in all the test environments (found at the apex of sector) and it is high yielder with broad adaptation which can be suggested to be best genotype for wider production. This is agreeing with the claim of [27] that if all the environment markers fall into one sector; this indicates that a single cultivar has the highest yield in all environments. Conversely, if environment markers fall into different sectors, it indicated that different cultivars superior in various sectors.

Regarding the genotype and environment evaluations based on biplot analysis, [27] stated that genotypes that have a small vector distance from the centre of the biplot are considered as stable genotypes. The authors also explained that the length of the environmental vector showing the magnitude of the influence of genotypic factors, the environments and their interactions. In this study, Arba Minch locations in both years (AM1 and AM2) had relatively large PC1 scores that they better discriminated among genotypes for root yield. Conversely, Hawassa locations (HAW1 and HAW2) had PC2 scores near zero, however; they were the worst performing environments for root yield in this study [Fig 1].

The mean yield versus stability view biplot was computed and presented based on ten OFSP genotypes tested at six distinct environments (three locations over two years) as depicted in [Fig 2]. The average environment coordinate (AEC) abscissa (the vertical line that passes through the biplot origin) divides genotypes into two groups: high yielders that yield more than the overall mean (right hand side) and lower mean yielders that yield less than the overall mean (left hand side). In this study, genotype G7 was the most stable with above average yield followed by genotypes G5 & G10. Genotypes G6, G8 and G9 were also the most stable genotypes but with the least yield performance (Fig 2). On the other hand, the genotype G3 was less stable as compared to all genotypes except G1. Although the G3 is less stable as compared to the other genotypes, it is the highest yielder variety in almost all the environments (across locations and years) and did not show specific adaptation to only one or two environments. Therefore, it can be suited for adaptation to wider areas.

**GGE-Biplot-based evaluation of test environments for representativeness and discriminating ability**

Based on explanation by [27], environments that have high PC1 scores are better at differentiating between genotypes, whereas those with low PC2 values are more representative of the average. The authors also proposed using a biplot to determine the representativeness of a test site, using the average environment as the reference. The average environment coordinate (AEC) abscissa was determined by averaging PC1 and PC2 values across all environments. The proximal angle with the AEC defines the representativeness of an individual location; the smaller the angle between the location vector and the AEC, the more representative the tested site. Based on the above fact, in this study, the two environments denoted as HAW1 (Hawassa year 1) and HAW2 (Hawassa year 2) were the most representative, followed by KOK1 (Koka year 1), while KOK2 (Koka year 2), AM2 (Arba Minch year 2), and AM1 (Arba Minch year 1) were the least representative in root yields of the tested genotypes [Fig 3]). As a result, two test environments, AM2 and AM1, are better distinguished between the test genotypes in terms of root yield and may be better test locations than others, which is in lined with the earlier reports on sweetpotato genotypes evaluation for root yield and yield components by [26].

**Conclusion**

Developing early-maturing orange-fleshed sweet potatoes is critical for production and productivity, especially in areas where persistent moisture stress causes severe food shortages. The evaluation and selection of the best genotype for that area are of utmost importance. This study identified, genotype G3 (13NC9350A-9-3) as the highest yielder variety in almost all the environments (across locations and years). The computation from the GGE biplot also revealed that the G3 was the vertex genotype of the polygon that performed best Overall, this genotype performed consistently across locations, has a very good level of resistance to SPVD severity, has an acceptable level of beta-carotene and dry matter contents, matures in a three-month period, and can also be a suitable variety for intercropping and relay cropping systems in low-moisture stress areas. Therefore, it can be recommended for verification and release for wider production in the country.

**Acknowledgements**

This work was supported by the Ethiopian Institute of Agricultural Research (EIAR) and sub-grants from SweetGAINS project of CIP (INV-002961), funded by the Bill and Melinda Gates Foundation. The authors duly acknowledged both funders for their financial support to run the field works

**References**

1. Ginting E. CAROTENOID EXTRACTION OF ORANGE-FLESHED SWEET POTATO AND ITS APPLICATION AS NATURAL FOOD COLORANT. Jurnal Teknologi dan Industri Pangan. 2013; 24:81–88. doi: 10.6066/jtip.2013.24.1.81.
2. Alam M, Rana Z, Islam S. Comparison of the Proximate Composition, Total Carotenoids and Total Polyphenol Content of Nine Orange-Fleshed Sweet Potato Varieties Grown in Bangladesh. Foods. 2016; 5:64. doi: 10.3390/foods5030064.
3. Belesova K, Agabiirwe CN, Zou M, Phalkey R, Wilkinson P. Drought exposure as a risk factor for child undernutrition in low- and middle-income countries: A systematic review and assessment of empirical evidence. Environment International. 2019; 131:104973. doi:10.1016/j.envint.2019.104973.
4. Ritchie H, Roser M. Micronutrient Deficiency. Our World in Data. 2024. Available from: https://ourworldindata.org/micronutrient-deficiency.
5. Staff Guidance Note on Macro prudential Policy - Detailed Guidance on Instruments. IMF Policy Paper. 2014. Available from: <https://doi.org/10.5089/9781498342605.007>.
6. Gurmu F, Hussein S, Laing M. The Potential of Orange-Fleshed Sweet Potato to Prevent Vitamin A Deficiency in Africa. International Journal for Vitamin and Nutrition Research [Internet]. 2014;84:65–78. doi: 10.1024/0300-9831/a000194.
7. Gurmu F, Mekonen S. Evaluation of root yield performance of newly bred orange-fleshed sweet potato genotypes in Ethiopia. Journal of Agricultural and Crop Research. 2019; 7:9–17. doi: 10.33495/jacr_v7i1.18.154.
8. Maquia I, Muocha I, Naico A, Martins N, Gouveia M, Andrade I, Goulao LF, Ribeiro AI. Molecular, morphological and agronomic characterization of the sweet potato (Ipomoea batatas L.) germplasm collection from Mozambique: Genotype selection for drought prone regions. South African Journal of Botany. 2013; 88:142–151. doi: 10.1016/j.sajb.2013.07.008.
9. Andrade MI, Naico A, Ricardo J, Eyzaguirre R, Makunde GS, Ortiz R, Grüneberg WJ. Genotype × environment interaction and selection for drought adaptation in sweetpotato (Ipomoea batatas [L.] Lam.) in Mozambique. Euphytica. 2016; 209:261–280. doi: 10.1007/s10681-016-1684-4.
10. HwARC (Hawassa Agricultural Research Center). 2015. Sweetpotato production and field management in Ethiopia, Production Manual. Hawassa Agricultural Research Center, Hawassa, Ethiopia
11. Grüneberg W, Eyzaguirre R, Diaz F, Boeck D, Espinoza J, Swanckaert JProcedures for the evaluation of sweetpotato trials. Manual. Procedures for the Evaluation of Sweetpotato Trials Manual.2019. <https://doi.org/https://doi.org/10.4160/9789290605225>.
12. Cox DF, Gomez KA, Gomez AA. Statistical Procedures for Agricultural Research. Journal of the American Statistical Association. 1985;80:486. doi: 10.2307/2287932.
13. Yan W, Hunt LA, Sheng Q, Szlavnics Z. Cultivar Evaluation and Mega-Environment Investigation Based on the GGE Biplot. Crop Science. 2000; 40:597–605. doi: 10.2135/cropsci2000.403597x.
14. Yan W. GGEbiplot—A Windows Application for Graphical Analysis of Multi-environment Trial Data and Other Types of Two‐Way Data. Agronomy Journal. 2001; 93:1111–1118. doi: 10.2134/agronj2001.9351111x.
15. Yan W, Tinker NA. Biplot analysis of multi-environment trial data: Principles and applications. Canadian Journal of Plant Science. 2006; 86:623–645. doi: 10.4141/p05-169.
16. Egea-Gilabert C, Pagnotta MA, Tripodi P. Genotype × Environment Interactions in Crop Breeding. Agronomy. 2021; 11:1644. doi: 10.3390/agronomy11081644.
17. Farshadfar E, Sutka J. Biplot analysis of genotype-environment interaction in durum wheat using the AMMI model. Acta Agronomica Hungarica. 2006; 54:459–467. doi: 10.1556/aagr.54.2006.4.8.
18. Mekonen S, Gurmu F, Getachew G. Reaction of introduced orange fleshed sweetpotato (Ipomoea batatas L.) genotypes for virus disease resistance and storage root yield performance under field conditions in Dilla and Hawassa, southern Ethiopia. Indian Phytopathology. 2023; 76:243–259. doi:10.1007/s42360-022-00590-w.
19. Rukundo P, Shimelis H, Laing M, Gahakwa D. Combining Ability, Maternal Effects, and Heritability of Drought Tolerance, Yield and Yield Components in Sweetpotato. Frontiers in Plant Science. 2017; 7. doi: 10.3389/fpls.2016.01981.
20. Gasura E, Mukasa S. Prevalence and implications of sweetpotato recovery from sweet potato virus disease in Uganda. African Crop Science Journal. 2011; 18. doi: 10.4314/acsj.v18i4.68648.
21. Ngailo S, Shimelis H, Sibiya J, Mtunda K, Mashilo J. Genotype-by-environment interaction of newly-developed sweet potato genotypes for storage root yield, yield-related traits and resistance to sweet potato virus disease. Heliyon. 2019;5:e01448. doi: 10.1016/j.heliyon.2019.e01448.
22. Shumbusha D, Ndirigwe J, Kankundiye L, Musabyemungu A, Gahakwa D, Ndayemeye PS, Mwanga ROM. ‘RW11-17’, ‘RW11-1860’, ‘RW11-2419’, ‘RW11-2560’, ‘RW11-2910’, and ‘RW11-4923’ Sweetpotato. HortScience. 2014; 49:1349–1352. doi: 10.21273/hortsci.49.10.1349.
23. Solárová J. Gardner, F. P., Pearce, R. B., Mitchell, R. L. (ed.): Physiology of Crop Plants. Biologia Plantarum. 1987; 29:21–21. doi: 10.1007/bf02902309.
24. Burgos B, Carpio R, Sanchez C, Paola S, Eduardo P, Espinoza J, et al., editors. A color chart to screen for high β-carotene in OFSP breeding. 15th Triennial Symposium of the International Society for Tropical Root Crops, Lima, Peru; 2009.
25. Rodrigues NR, Barbosa JL, Barbosa MIMJ. 2016. Determination of physico‐chemical composition, nutritional facts and technological quality of organic orange and purple‐fleshed sweet potatoes and its flour. International Food Research Journal; 2016; 23:2071-2078.
26. Gurmu F. Stability Analysis of Fresh Root Yield of Sweetpotato in Southern Ethiopia using GGE Bi-Plot. International Journal of Pure Agricultural Advances. 2017; 1:1–9. doi: 10.20448/813.11.1.9.
27. Yan W, Kang MS, Ma B, Woods S, Cornelius PL. GGE Biplot vs. AMMI Analysis of Genotype‐by‐Environment Data. Crop Science. 2007; 47:643–653. doi: 10.2135/cropsci2006.06.0374.

**Figures**

**Fig 1. Showing the “which-won-where” pattern of tested genotypes for total root yield performance across locations over years based on genotype plus genotype-by-environment (GGE) biplot. Where, HAW1=Hawassa year 1; HAW2=Hawassa year 2; Kok1 = Koka year 1; Kok2 = Koka year 2; AM 1 = Arba Minch year 1; AM2= Arba Minch year 2**

Fig 2. The mean performance and stability of 10 evaluated orange-fleshed sweetpotato genotypes in six environments

**Fig 3. The vector view of GGE biplot of 10 OFSP genotypes evaluated across locations over years**

**Supporting information**

**S1 Data. Weather information for the testing locations in 2021 and 2022**

**S1 Table. Description of experimental materials used for the study**
