# Supplementary material for: Evaluation of the performance and stability of early maturing orange-fleshed sweetpotato genotypes in selected areas in Ethiopia
Source: PLoS One. 2024 Oct 25;19(10):e0310273. doi: 10.1371/journal.pone.0310273 (PMC11508074; doi:10.1371/journal.pone.0310273)
Supplement: Attachment — Submitted filename: PLOS ONE Review Report.docx [file pone.0310273.s005.docx]

**PLOS ONE: Review Report PONE-D-23-35204R1 - [EMID:a610d6319ebd3229]**

1. The study presents the results of original research.

- Yes.

2. Results reported have not been published elsewhere.

- Yes

3. Experiments, statistics, and other analyses are performed to a high technical standard and are described in sufficient detail.

- Authors should discuss their results in details. Their discussion is very shallow.

4. Conclusions are presented in an appropriate fashion and are supported by the data.

- Yes but should be improved

5. The article is presented in an intelligible fashion and is written in standard English.

- The English is not good. They should request assistance from someone who is good in written English.

6. The research meets all applicable standards for the ethics of experimentation and research integrity.

- Yes

7. The article adheres to appropriate reporting guidelines and community standards for data availability.

- Yes
