# Supplementary material for: Evaluation of the performance and stability of early maturing orange-fleshed sweetpotato genotypes in selected areas in Ethiopia
Source: PLoS One. 2024 Oct 25;19(10):e0310273. doi: 10.1371/journal.pone.0310273 (PMC11508074; doi:10.1371/journal.pone.0310273)
Supplement: Attachment — Submitted filename: Comments PONE-D-23-35204_R1.docx [file pone.0310273.s006.docx]

**Comments PONE-D-23-35204_R1**

The article "Evaluation of the performance and stability of early-maturing orange-fleshed sweetpotato genotypes in selected moisture stress areas in Ethiopia" has been thoroughly examined. In my opinion, there is a lack of clarity in the study and research completed for this article. It needs to be improved since most of the phrases are written in an exceedingly casual and cursory style, and they lack depth. I have noticed few major issues in the article and are as follows-

**Following points needs clarification and are as follows-**

1. The intricacy of the article cannot be adequately conveyed by the abstract and introduction. The Sweetpotato Virus Disease (SPVD) was graded, yet there is a lack of information in the introduction. The introduction has not addressed root qualities, their function, and a rational debate of drought tolerance to enhance comprehension. The term "drought" is frequently used in the article because it is a challenging parameter to measure, usually using physical instruments and techniques that require specialized knowledge.

2. The current study used nine genotypes, which is a smaller number. It's unclear which check is being used. Check-related details ought to have been expanded upon.

3. Instead of using "drought and moisture stress condition," I advise the authors to use the phrase irregular rainfall or inadequate rainfall in entire manuscript.

The goal of the study, which I believe may not be achieved by carrying out the research, is to find early-maturing genotypes or varieties that may mature early and avoid the consequences of low moisture stress brought on by protracted periods of drought.

4. It is necessary to include more details regarding the genotypes of sweet potatoes that mature early and late in the article as well as in introduction.

5. There may be a need to improve the disease screening procedures because non-significant differences in SPVD were observed across locations.

6. While the genotype that was identified may be useful in the local context, it may not be of interest to breeders of sweet potatoes internationally.

As a result, the article needs major revisions and extra editing. It cannot be approved for publishing in its current state.
