# Supplementary material for: Evaluation of the performance and stability of early maturing orange-fleshed sweetpotato genotypes in selected areas in Ethiopia
Source: PLoS One. 2024 Oct 25;19(10):e0310273. doi: 10.1371/journal.pone.0310273 (PMC11508074; doi:10.1371/journal.pone.0310273)
Supplement: Attachment — Submitted filename: Response to reviewers comments 28072024.doc [file pone.0310273.s007.doc]

**A letter for responding to reviewers comments**

**Date 28/07/2024**

**To: Academic Editor**

**PLOS ONE**

Subject: **Manuscript Ref: PONE-D-23-35204R1**

**Dear Dr Jiban Shrestha,**

Thank you for allowing us to resubmit a revised version of our research paper titled "**Evaluation of the performance and stability of early maturing orange-fleshed sweetpotato genotypes in selected areas of Ethiopia**" to the PLOSONE journal. We appreciate the time and efforts you and the reviewers have put into providing important feedback on our manuscript. We are grateful to the reviewers for providing valuable feedback on our paper. We were able to apply improvements that addressed the majority of the reviewers' comments. In addition, almost all sections of the manuscript were reshaped and improvements have been made accordingly to better reflect the manuscript's content. The abstract, introduction, results and discussion, conclusion and references were revisited and substantially updated, as well as the plosone formatting and style.

The manuscript underwent a rigorous review with the assistance of three senior research scholars.

1. **Dr. Fekadu Gurmu** (fekadugb@gmail.com), Cell phone: +251911743625, senior plant breeder and geneticist, Ethiopian Institute of Agricultural Research, Addis Ababa, Ethiopia
2. **Dr. Zerihun Yemataw** ([zerihunyemataw2@gmail.com](mailto:zerihunyemataw2@gmail.com)), Cell phone: +251911960755, senior plant breeder and geneticist, Sidama Agricultural Research Institute, Hawassa, Ethiopia
3. **Mr. Melese Lemma** ([meleselema72@gmail.com](mailto:meleselema72@gmail.com)), Cell phone:+251910057232, Senior plant breeder and geneticist, Sidama Region Agricultural Research Institute (SIRARI), Hawassa, Ethiopia

***Here is a point-by-point response to the reviewers’ comments and concerns.***

**Additional Editor Comments:**

The authors need to include all suggestions given by reviewers.

**Response**: We agreed to apply all the given suggestions and comments. Thank you

**Reviewer #1: The authors have effected the necessary corrections; the manuscript should therefore be accepted for publication**

**Response**: Thank you

**Reviewer #3: The article lacks detailed discussion in terms of comparison with past related studies done by other researchers. Hence they should improve discussion of their results. They should improve their English.**

**Response:** For all the above concerns, it was agreed to take action. Thank you for pointing this out. As a result, we thoroughly reviewed the entire manuscript, and we made corrections by comparing the current findings with recently published works. Also given suggestions including improving English were effected. These corrections have been indicated with a track changer in the text document of the manuscript. Also, clean copy (without a track changer) is provided during submission.

**Reviewer #4: Following points needs clarification and are as follows-
1. The intricacy of the article cannot be adequately conveyed by the abstract and introduction. The Sweetpotato Virus Disease (SPVD) was graded, yet there is a lack of information in the introduction. The introduction has not addressed root qualities, their function, and a rational debate of drought tolerance to enhance comprehension. The term "drought" is frequently used in the article because it is a challenging parameter to measure, usually using physical instruments and techniques that require specialized knowledge.**

**Response:** [Corrective action is agreed upon]. We have highlighted the Sweetpotato Virus Disease (SPVD) and the impact that it has on yield reduction. The information is included in the introduction section. Concerning the usage of the term "drought,” it was related in terms of erratic rainfall situations and their causes. But we have agreed on the suggestions given by reviewers, and we are glad of that. ***In agricultural terms, drought is defined as when the amount of moisture in the soil no longer meets the needs of a specific crop in order to achieve the highest yield.* However,**

We corrected word to erratic/irregular rainfall throughout the document. Thus, we have made a lot of amendments to the text of the document. The changes were indicated on the track changer in red font.

**2. The current study used nine genotypes, which is a smaller number. It's unclear which check is being used. Check-related details ought to have been expanded upon.**

**Response: concerning the question raised about the small number of genotypes.**

**Initially, over 400 genotypes were characterized based on various traits (root yield and yield-related traits, SPVD reaction). When the disease signs appeared at each evaluation step, genotypes exhibiting disease symptoms were discarded from each family. Finally, genotypes that demonstrated better performances for the traits of interest were advanced to the preliminary trial. Out of these 25 genotypes, nine were promoted to a multi-environment trial (NVT) in order to evaluate their performance across diverse locations over the years. Thus, these materials came from various selection stages.**

**3. Instead of using "drought and moisture stress condition," I advise the authors to use the phrase irregular rainfall or inadequate rainfall in entire manuscript. The goal of the study, which I believe may not be achieved by carrying out the research, is to find early-maturing genotypes or varieties that may mature early and avoid the consequences of low moisture stress brought on by protracted periods of drought**.

**Response**: Agreed, and we have made a lot of amendments, which are indicated with a track changer in the body of the document. Furthermore, concerning the goal of this study, it is known that sweet potato can be considered a drought-tolerant crop, but it suffered low moisture stress for a prolonged period, including the root initiation period. Early-maturing varieties (3–4 months) can escape the terminal drought due to the prolonged period of drought that we are facing now due to climate change. The details for the essence and strategies for early maturing varieties are also elaborated in the introduction section during these revisions.

**4. It is necessary to include more details regarding the genotypes of sweet potatoes that mature early and late in the article as well as in introduction**

**Response:** We have included the details as suggested by reviewer. Thank you.

**5. There may be a need to improve the disease screening procedures because non-significant differences in SPVD were observed across locations.**

**Response**: Agreed, and in the future we will come with an advanced protocol. We’re also expecting to use some molecular techniques to confirm the scored values since we have seen similar results, specifically for SPVD, in various stage trials.

**6. While the genotype that was identified may be useful in the local context, it may not be of interest to breeders of sweet potatoes internationally.**

**Response:** Agreed

With best regards,

Bililign Mekonnen Senior researcher and coordinator of national root crops research program in Ethiopia, Hawassa, Ethiopia
