# Supplementary material for: Evaluation of the performance and stability of early maturing orange-fleshed sweetpotato genotypes in selected areas in Ethiopia
Source: PLoS One. 2024 Oct 25;19(10):e0310273. doi: 10.1371/journal.pone.0310273 (PMC11508074; doi:10.1371/journal.pone.0310273)
Supplement: Attachment — Submitted filename: Manuscript-plosone-Author editted clean copy- re.doc [file pone.0310273.s009.doc]

Evaluation of the performance and stability of early maturing orange-fleshed sweetpotato genotypes in selected areas in Ethiopia

**Bililign Mekonnen^1*^, Fekadu Gurmu^2^**

**1** Sidama Region Agricultural Research Institute (SIRARI), Hawassa Agricultural Research Centre, Hawassa, Ethiopia, **2** Ethiopian Institute of Agricultural Research (EIAR), Addis Ababa, Ethiopia

* bililign.m@gmail.com

# Abstract

Development of orange-fleshed sweetpotato varieties for short-term harvesting is crucial to ensuring food and nutrition demands in mid- to low-land areas with erratic rainfall. A National Variety Trial was conducted in 2021 and 2022 during the main cropping season using ten OFSP genotypes in three locations in the Sidama, Southern, and Oromia regions of Ethiopia, namely Hawassa, Arbaminch, and Koka. The objective was to develop and select early-maturing and high-yielding genotypes for short-term harvesting (3–4 months). The trial was conducted in a randomized complete block design with three replications. Data were collected on root yield and yield-related traits, sweetpotato virus disease reactions (SPVD), root dry matter (DMC), and beta-carotene contents (BCC) and subjected to analysis of variance. A genotype plus genotype by environment interaction (GGE) bi-plot was also used to determine genotype stability. The results revealed that differences observed across locations and among genotypes were highly significant (p<0.001), reflecting the existence of differential responses among genotypes in varied environments. Combined analysis showed that G3 (13NC9350A-9-3) outperformed the other genotypes for most of the traits evaluated, with the exception of DMC (which is equivalent to the check variety Alamura), with a yield advantage of 41.4% over the check. The GGE biplot also confirmed that the G3 was the vertex genotype of the polygon that performed best. It also performed consistently in all environments. It had a score of 1.39, with 1-9 scoring method, indicating it falls within the resistant range, had optimal levels of BCC (5.5 mg/100 g) and DMC (30.0%), including early maturations, and can be a suitable variety for areas where irregular rainfall disrupts crop production. Therefore, based on its overall performance, G3 (13NC9350A-9-3) is recommended for verification and release to cultivate in mid-to low-land areas where rainfall is erratic for the production of sweetpotatoes in Ethiopia.

**Introduction**

Sweetpotato is one of the highly valuable root crops that can best adapt to diverse agro-ecological conditions and thrive in areas where rainfall is erratic. Sweetpotato plays a crucial role in the global food system, addressing food needs, reducing poverty, and enhancing food security [1]. The orange-fleshed sweetpotato group has an advantage over the white sweetpotato group in terms of providing a healthy and nutritious food for areas where food and nutritional security are key challenges [2, 3]. Globally, sweetpotato ranks seventh in production, after rice, wheat, maize, potato, barley, and cassava, with an annual yield of around 119 million metric tons [4]. In Africa, it covered 4.21 million hectares of land, producing around 29.11 million tones [4]. In Ethiopia, it was grown on approximately 62,115 hectares in Meher season, with a total production of more than 1.5 million tones, only Meher in season [5]. According to Central Statistical Agency (CSA) report [6], it is the 2^nd^ most important root crop next to Enset (*Ensete ventricosum*) crop in Ethiopia. More importantly, particularly OFSP varieties, has received a due attention as a means for nutrition-sensitive agriculture, emergency response mechanisms in areas with erratic rainfall, for food diversification strategies [7]. Nowadays an emerging issues such as recurrent drought caused by the occurrence of erratic rainfall/irregular rainfall, Vitamin A deficiency (VAD), a rapidly growing human population, susceptibility of the existing sweetpotato varieties to sweetpotato virus disease, and expansion of urbanization, call agricultural researchers to emphasize on the development of nutritious, high-yielding, and early-maturing OFSP varieties [8-10]. According to [11], in agricultural terms, drought is defined as when the amount of moisture in the soil no longer meets the needs of a specific crop in order to achieve the highest yield. Thus, it is also one of the major abiotic stressors that hinder crops from obtaining their potential maximum yield, including sweetpotato production. Although sweetpotato regarded as drought tolerant crop, the onset of drought (early, mid-season or terminal), as well as the intensity and duration, significantly affect how much storage root yield occurs. The genotype also plays a significant role and different sweet potato cultivars respond differently to quantities of moisture content of soil [12].

Another health concern is micronutrient deficiency, among which Vitamin A deficiency is one of the five major micronutrient mineral deficient nutrients, remains a serious health concern in resource-poor rural communities around the world [13, 14]. Various strategic approaches, viz., supplementation, fortification, diversification, and biofortification, are being recommended as possible avenues to curb malnutrition. Cultivation and consumption of biofortified crops commonly grown in an area is thought to be one of the most affordable strategies to address malnutrition-related issues. In this context, biofortified orange-fleshed sweetpotato cultivars play an important role in addressing vitamin A deficiency by serving as an easily affordable source of nutritious diet [15, 16]. The production and productivity of nutritious sweetpotato varieties can ensure the availability of foods for enhanced food system. However, cultivation is affected by several constraints that include both biotic and abiotic factors ranging from virus diseases, fungal diseases, insect pests, and weeds, biotic factors such as declining soil fertility, recurrent drought, and socioeconomic factors associated with a lack of clean planting materials, insufficient availability of high yielding varieties, etc [17]. Among biotic factors, more than 20 virus diseases are known to infect cultivated sweetpotatoes worldwide [18], specifically, sweetpotato virus disease (SPVD) which is occurred by co-infection of sweetpotato feathery mottle virus (SFMV) and sweetpotato chlorotic stunt virus (SPCSV) is a devastating disease worldwide [19,20].

According to [21], the degree of yield loss to virus infection varies based on the resistance levels of the sweet potato genotypes, the crop's growth stage at which the diseases occur, and the agro-ecology of the country. In different regions of the world, the amount of yield loss or damage caused by SPVD varies from 50 to 100% [19, 22]. In Ethiopia, the synergistic infection of SPVMV and SPCSV resulted in a yield loss of up to 37% [23]. When it comes to managing plant diseases such as SPVD, the development and use of resistant/tolerant genotypes is the best option in terms of efficacy, sustainability, eco-friendliness, and low cost of production [24,25] .

Additionally, following the global climate change driven moisture stress caused by irregular rainfall is currently threatening sustainable crop production and posing existential food problems to smallholder farmers [11]. This problem is currently affecting sweetpotato production and productivity in Ethiopia. Sweetpotato varieties that mature early are a better resort to this challenge. However, the response of these varieties depends on factors like the onset and severity of droughts due to irregular rainfall [26].

One of the approaches to addressing the consequences associated with low moisture stress caused by erratic rainfall is the development of early-maturing varieties, which have the advantage of early bulking and maturation in a short period of time, i.e., harvesting between 3-3.5 months. Sweetpotato varieties in Ethiopia are categorized into three groups based on maturity level. The first groups are early maturing, taking 90 to 120 days, the second groups are medium maturing, taking 121 to 150 days, and the third groups are late maturing, taking more than 150 days. Early maturing groups are recommended for lowland areas characterized with erratic /irregular rainfall, while medium and late maturing groups are recommended for mid-altitude areas with medium to high rainfall during the growing season [27]. Accordingly, early maturing varieties have an advantage over medium to late maturing varieties in terms of producing food in a short period of time with a limited amount of available rainfall eventually ensuring food availability for needy areas.

Although sweetpotatoes are known to be able to adapt to a wide range of agro-ecological zones, several authors have reported the existence of varietal differences due to its sensitivity to genotype-environment interaction [28-31]. The authors explained how sweetpotato genotypes are affected by genotype-environment interaction (GEI) and how stable they are in different ecological conditions across their respective countries. In this context, one of the well-known statistical methods that helps crop variety selection and recommendation in various environments is genotype-by-environment interaction (GGE) biplot analysis, which quantifies genotype-by-environment interaction (GxE) effect based on multi-location trials [32, 33].

Based on the above rationale, (deleted since this part is repeated in the heading of ‘description of experimental materials’) a national variety trial was conducted over two years (2021-2022) at three locations (Hawassa, Koka, and Arbaminch) using advanced ten genotypes (nine selected, one check) with the objective of identifying early-maturing varieties that can mature early and withstand more prolonged low moisture stress caused by irregular or erratic rainfall.

**Materials and methods**

##  Description of study areas and its weather conditions

The trial was conducted for two consecutive years (2021 and 2022) at three locations namely Hawassa, Arbaminch, Koka in Sidama, South, and Oromia regions, respectively, in Ethiopia. In terms of meteorological conditions during the sweetpotato growing season in 2021, Arbaminch, Hawassa, and Koka areas received 541.2, 993.5, and 523.6 mm of rain, respectively. However, the growing season rainfall was only 291.2, 601 and 470 mm in 2022, in that respective order. This showed that sweetpotato plants received lower amount of rainfall in 2022 than 2021; and the rainfall received during the growing season was the lowest at Arbaminch, intermediate at Koka and the highest at Hawassa [S1 Data]. As a result of inconsistent rainfall, three months of low moisture stress were experienced in 2022. In October and September, Arbaminch and Koka received more rainfall than Hawassa did. Furthermore, whether the year was good or bad, rainfall in August and September was always less than what was needed for crops in Arbaminch, as it was in October and November in Koka. In Hawassa, August and September were occasionally drier [S1 Data].

**Testing sites permission**

This work is a part of the Ethiopian Institute of Agricultural Research (EIAR). The institute already had linkages with regional agricultural research centers and agricultural universities in Ethiopia to collaborate on any research activities. Furthermore, when designing field experiments, any field research activities must go through a review process that includes testing locations. The activity can be carried out at the designated locations (sites) without any limitations or requests for site authorization once it has successfully completed this process.

**Description of experimental materials**

Botanical seeds derived from 16 polycross families were introduced from Uganda in 2016. Prior to establish field trial, over 400 botanical seeds of all families were characterized based on heritable traits such as root yield, reaction to disease and insect pests. More importantly, genotypes with the best performance including reaction to sweetpotato virus disease and insect pests were advanced to the next yield performance testing, whereas those with diseases symptoms in each family discarded. Consecutively, genotypes with better performance for afro-mentioned traits, i.e., nine selected genotypes and one standard check, which was released in 2019 and is currently being grown in major sweetpotato growing areas in Ethiopia, were tested in a national variety trial at three locations (Hawassa, Koka, and Arbaminch) over a two-year period (2021-2022) following variety releasing requirements in Ethiopia, as shown in [S1 Table].

**Field design and management**

The field experiment was conducted in a Randomized Complete Block Design (RCBD) replicated thrice. Each genotype is planted on a plot size of 2.4 m wide and 3 m length (a total plot area of 7.2 m^2^). Each plot consisted of four rows/ridges, each row containing 10 plants/row and a total of 40 plants/cuttings/plot. The spacing between plants and rows were 0.3 m and 0.6 m, respectively. In each plot, one plant (vine cutting) of 30 cm length with 5-8 nodes was planted in the prepared holes by burying one-third of the plant in the soil. After one week, dead plants (vine cuttings) were replaced by new ones (to maintain uniformity of plant population per plot), and after the fourth week, earthening was done, and all plots were manually weeded according to the recommendation for sweetpotato production in Ethiopia [34].

**Data collection**

All root yield and root-yield related traits were collected on a plot basis from the two middle rows, with the exception of the two plants grown at each ends of the row and the two border rows. Data on SPVD severity was collected prior to one month of harvesting, whereas data on root yield and yield-related traits were recorded during harvest [35]. Data for root dry matter content (DMC) were determined using the procedures suggested by [36, 37], with minor variations. A sample of four roots from each plot was collected. For each genotype in a replication, 200 g of intact roots were cut. The prepared samples were put in a paper bag and dried in an oven set to 70 °C for 72 hours, and recording was done until they maintained a constant weight. Finally, the dried samples were weighed using a sensitive balance or weighing scale, and the dry matter content was calculated as a percentage of the fresh weight. To estimate beta-carotene content (BCC), a color chart was used as suggested by [38].

**Statistical analysis**

**Analysis of variance**

An analysis of variance was conducted on all of the data collected. Data for each location were analyzed separately before performing a combined analysis to check the homogeneity of error variance across locations over years using the F-ratio, which was found to be non-significant (data not displayed here), and then the combined analysis was computed using SAS software version 9.3 [39]. The Fisher's protected least significant difference (LSD) test was used to compare the treatments at 1% and 5% probability levels following the procedure suggested by [40].

The following statistical model was used for combined analysis of variance:

$Y_{\text{ijkl}}=\mu+G_{i}+L_{j}+Yk+\text{GL}_{\text{ij}}+\text{GY}\text{ik}+\text{LY}\text{jk}+\text{GLY}\text{ijk}+B_{l(\text{jk})}+\varepsilon_{\text{ijkl}}$

Where: Y_ijkl_ is observed value of genotype *i* in block *l* nested in (location *j* and year *k)*, μ is grand mean, G_i_ is genotype effect, L_j_ is location effect, Y_k_ is year effect, GL_ij_, GY_ik_ and GLY_ijk_ are the interaction effect of genotype *i* with location *j*, genotype *i* with year k and genotype *i* with location *l* and year *k*, є_ijkl_ is error (residual) effect.

**Genotype by environment interaction (GEI) analysis**

The data were analyzed in GenStat (18^th^ version) for GEI and stability of genotypes using the GGE biplot procedure [41-43]. The following model for the GGE biplot based on singular value decomposition (SVD) of the principal components was used:

$$\bar{Y}_{\text{ij}}-\mu_{i}-\beta_{j}=\sum\lambda_{k}\alpha_{\text{ik}}\gamma_{\text{jk}}+\varepsilon_{\text{ij}}$$

Where: $\bar{Y}_{\text{ij}}$is the performance of genotype *i* in environment *j*, $\mu$ is the grand mean, $\beta_{j}$ is the main effect of environment *j*, *k* is the number of principal components (PC); $\lambda_{k}$is singular value of the *k*^th^ PC; and $\alpha_{\text{ik}}$and$\gamma_{\text{jk}}$are the scores of *i*^th^ genotype and *j*^th^environment, respectively for PC_k_; $\varepsilon_{\text{ij}}$ is the residual associated with genotype *i* in environment *j*.

**Results and discussion**

**Analysis of variance of root yield and yield-related traits**

A combined analysis of variance revealed the presence of a highly significant difference (p<0.001) among tested genotypes for all the traits considered [Table 1]. There was also a significant effect (p<0.001) of location by genotype interaction for all traits except SPVD, the differences observed indicating that genotypes tested responded differently to the test locations.

The presence of significant genotype-location interaction is a clear indicator of genotypic differences and their sensitivity to various locations, which ultimately complicates the process of variety recommendation to wider areas [44]; hence, demanding further stability analysis to identify a stable genotype across locations as is essential suggested by [45]. The three-way interaction effect (genotype x location x year) was significant for all traits considered in this study, reflecting that the observed significant differences could be associated with genotypic differences, growing season, cultural management, and environmental conditions where the trial was conducted. The non-significant differences observed among evaluated genotypes for SPVD disease reaction may be related to some factors, such as unfavorable environmental conditions for disease development, including the absence of vectors that carry and spread virus diseases from plant to plant by sucking insects in the trial areas. This agrees with the earlier suggestion given by [21].

Table 1. Analysis of variance for various traits of ten OFSP genotypes evaluated at three locations over two years

| Source of variance | D.F | Mean squares | | | | | | |
| --- | --- | --- | --- | --- | --- | --- | --- | --- |
|  |  | SPVD  (1-9) | RL  (cm) | RG  (cm) | AGFW  (t ha^-1^) | TRYLD  (t ha^-1^) | DMC  (%) | BCC  (mg100g-1) |
| Rep (LxY) | 10 | 0.33^ns^ | 67.15*** | 16.00** | 743.50*** | 6.17^ns^ | 0.0002^ns^ | 14.10 ^ns^ |
| Genotype (G) | 9 | 3.37*** | 126.47*** | 59.65*** | 592.64*** | 1807.89*** | 0.09*** | 26.21*** |
| Location (L) | 2 | 1.59^ns^ | 1135.15*** | 2893.19*** | 25839.80*** | 1632.88*** | 0.06*** | 19.20** |
| Year (Y) | 1 | 10.58*** | 50.47** | 61.34*** | 870.58** | 13.68^ns^ | 0.0003^ns^ | 22.31** |
| GxL | 18 | 3.43*** | 28.86*** | 12.94** | 1075.73*** | 269.75*** | 0.06*** | 19.32** |
| GxY | 9 | 2.30** | 3.47^ns^ | 15.02** | 152.00^ns^ | 31.95*** | 0.0002^ns^ | 21.30** |
| LxY | 2 | 17.35*** | 12.62^ns^ | 19.51** | 15925.83*** | 462.35*** | 0.08*** | 23.00** |
| GxLxY | 18 | 1.57** | 5.93^ns^ | 15.31** | 142.64^ns^ | 76.95*** | 0.0002^ns^ | 20.21** |
| Error | 108 | 0.73 | 5.41 | 6.00 | 175.87 | 5.17 | 0.0002 | 4.1 |

Where, 1-9 scale (1 = immune, 9 =Susceptible, hence 1-3 = resistant, 4–6 = medium and 7-9 = susceptible), D.F=degree of freedom, RL=Root length, RG=Root girth, AGFW=Above-ground fresh weight, TRYLD=Total root yield, DMC=Dry matter content, BCC=Beta-carotene content.

**Performance of genotypes for mean root yield in test environments**

The root yield trait of genotypes evaluated differed significantly across test locations over the years [Table 2]. The differences observed among the evaluated genotypes could be attributed to genetic variations inherent in each genotype, which resulted in varied responses when evaluated in different locations [16]. In year 1 (2021), the mean root yield of genotypes ranged from 6.4 t ha^-1^ for genotype G9 (CORDNER-15-2) to 37.0 t ha^-1^ for genotype G3 (13NC9350A-9-3). In year 2 (2022), for the same genotypes, G9 and G3, root yields ranged from 7.0 t ha^-1^ to 35.5 t ha^-1^, respectively, with an overall mean of 18.3 t ha^-1^ in this study. In terms of each location performance, the highest root yield was recorded for G3, followed by G1 (MUSG014052-51-5) with values of 49.0 and 48.9 t ha^-1^, respectively, from the Arbaminch location in year 1. In year 2, the highest root yield of 42.5 t ha^-1^ for G3 was obtained from Koka location. In both years, genotype G3 was the highest performer over the stand-check variety, with a yield advantage of 41.4%. Two locations, namely Koka and Arbaminch, can be considered the best environment over Hawassa locations, as confirmed by the genotypes that perform the best in this study. Among the ten evaluated genotypes, genotype G3 produced the highest root yield (t ha^-1^) in all environments (locations vs. years) [Table 2]. The five genotypes G4 (CN1448-49-26-12), G6 (107031-18-5), G7 (105413-5 (Pl. check)), G8 (105413-13), and G9 (CORDNER-15-2) gave the lowest root yields, i.e., <10 t ha^-1^, reflecting their poor performance or very low yields under the evaluated environmental conditions. When evaluating and selecting sweetpotato genotypes for areas where irregular rainfall poses a challenge for the production of crop varieties, it is crucial to consider genotypes that perform better for combined traits such as early maturation and high root yield for further recommendation [46].

Table 2. Root yield (t ha^-1^) performance of OFSP genotypes evaluated across locations and over years

| **Genotype code** | Genotype name | **2021** | | | | **2022** | | | | **Genotype by Environment** | **Yield advantage over standard check (%)** |
| --- | --- | --- | --- | --- | --- | --- | --- | --- | --- | --- | --- |
|  |  | **Hawassa** | **Koka** | **ArbaMinch** | **Mean** | **Hawassa** | **Koka** | **ArbaMinch** | **Mean** |  |  |
| G1 | MUSG014052-51-5 | 14.0 | 13.5 | 48.9 | 25.5 | 13.7 | 8.7 | 32.6 | 18.3 | 21.9 | -14.5 |
| G2 | MUSG014001-3-7 | 10.7 | 15.9 | 30.6 | 19.0 | 12.9 | 17.4 | 20.4 | 16.9 | 18.0 | -29.7 |
| G3 | 13NC9350A-9-3 | 33.7 | 28.5 | 49.0 | 37.0 | 30.3 | 42.5 | 33.6 | 35.5 | 36.2 | 41.4 |
| G4 | CN1448-49-26-12 | 7.0 | 12.5 | 8.0 | 9.2 | 6.9 | 20.2 | 7.3 | 11.5 | 10.3 | -59.8 |
| G5 | CN1448-49-28-9 | 11.6 | 26.4 | 32.3 | 23.5 | 15.3 | 30.0 | 28.3 | 24.6 | 24.0 | -6.3 |
| G6 | 107031-18-5 | 7.7 | 8.5 | 4.7 | 7.0 | 8.1 | 6.5 | 7.4 | 7.3 | 7.1 | -72.3 |
| G7 | 105413-5 | 9.9 | 22.2 | 41.1 | 24.4 | 16.2 | 31.2 | 27.4 | 24.9 | 24.7 | -3.5 |
| G8 | 105413-13 | 6.5 | 10.0 | 4.2 | 6.9 | 7.2 | 7.7 | 7.0 | 7.3 | 7.1 | -72.3 |
| G9 | CORDNER-15-2 | 6.4 | 9.60 | 3.1 | 6.4 | 6.1 | 7.8 | 7.0 | 7.0 | 6.7 | -73.8 |
| G10 | Alamura (Check) | 13.2 | 23.4 | 41.3 | 25.9 | 14.8 | 36.3 | 24.6 | 25.2 | 25.6 | - |
| Mean | | 12.0 | 17.1 | 26.3 | 18.8 | 13.1 | 21.0 | 19.6 | 17.7 | 18.3 |  |
| LSD (5%) | | 3.7 | 4.2 | 4.8 | 4.2 | 3.4 | 3.9 | 3.5 | 3.6 | 3.9 |  |
| CV (%) | | 18.3 | 14.6 | 11 | 14.6 | 15.5 | 11.1 | 10.6 | 12.4 | 13.5 |  |

**Mean performance of genotypes for root yield-related traits and SPVD reaction**

A combined analysis of variance revealed the presence of a significant difference (p<0.05) among genotypes in response to SPVD. The severity of symptoms for the evaluated genotypes ranged from 1.39 for G3 (13NC9350A-9-3) to 2.78 for G5 (CN1448-49-28-9), on a 1–9 rating scale, with an overall mean of 1.94 [Table 3]. The results showed that the majority evaluated genotypes had a high level of tolerance/resistance to SPVD, with a low score below 3.0, falling within the range of resistance to SPVD severity [21, 35]. The low level of disease severity among evaluated genotypes for SPVD, which indicates the presence of an unsuitable environment for the virus to evolve, as well as the lack of vectors or carriers that spread the virus from plant to plant near experimental sites [20, 47]. While selecting and advancing sweetpotato genotypes for later stages, it is suggested to assess their sensitivity to SPVD reactions along with other desirable traits [20, 21]. The significant differences (p<0.05) were observed in root length (cm) trait. The root length varied from 7.98 cm to 15.10 cm for genotypes G8 (105413-13) and G3 (13NC9350A-9-3), respectively, with an overall mean of 11.78 cm (Pl. check with table). Root girth trait ranged 5.39 cm to 10.41 cm, respectively for G8 (105413-13) and G7 (105413-5) with overall mean of 7.96 cm [Table 3? (Pl. check)]. Root length and root girth traits are among the best traits in sweetpotato genotypes screening process as these two traits can be an indicator for earliness (early bulking). Preferred genotypes for market appeal include those with early bulking and roots of appropriate size, i.e., root length no longer than 15 cm, the smallest no less than 10 cm, and root-girth/diameter at least greater than 4 cm; this is roughly what most consumers stated as their ideal root sizes, and it is now incorporated into the target product traits profile.

Genotypes varied significantly for above-ground fresh weight (t ha^-1^) in all environments, i.e., three locations vs. two years [Table 3]. The highest mean yield of 35.5 t ha^-1^ was recorded for G5 (CN1448-49-28-9) whereas the lowest mean yield of 25.2 t ha^-1^ recorded for check variety G10 (Alamura) (Please check with table values). Genotypes with combination of traits, such as high root yield and above-ground biomass yield (above-ground fresh weight) can be utilized for both human consumption and animal feed, particularly in areas under land constrain [48]. The harvest index (HI) performance of genotypes revealed significant variation. The genotype G3 (13NC9350A-9-3) had the highest mean value of 61% of HI, while the genotype G9 (CORDNER-15-2) had the lowest mean value of 20% of HI. A genotype with a high harvest index value is believed to have a high efficiency in storage root production in relationship to its biological yield. [49] reported that the harvest index signified the distribution of assimilation between economic and total plant biomass. The authors also explained that high yielding varieties have a higher harvest index than low yielding varieties in terms of fresh root yield.

Table 3. Mean performance of ten OFSP genotypes for various traits across locaction over years

| Code | Genotypes | SPVD  (1-9 scale) | RL  (cm) | RG  (cm) | AGFW  (t ha^-1^) | HI  (%) |
| --- | --- | --- | --- | --- | --- | --- |
| G1 | MUSG014052-51-5 | 1.83 | 11.84 | 8.67 | 37.19 | 43.00 |
| G2 | MUSG014001-3-7 | 1.61 | 13.67 | 7.43 | 32.80 | 43.00 |
| G3 | 13NC9350A-9-3 | 1.39 | 15.10 | 9.03 | 27.74 | 61.00 |
| G4 | CN1448-49-26-12 | 1.72 | 10.51 | 6.47 | 24.50 | 32.00 |
| G5 | CN1448-49-28-9 | 2.78 | 13.76 | 9.87 | 41.24 | 49.00 |
| G6 | 107031-18-5 | 2.11 | 9.71 | 5.98 | 30.73 | 25.00 |
| G7 | 105413-5 | 2.55 | 12.06 | 10.41 | 34.31 | 50.00 |
| G8 | 105413-13 | 1.78 | 7.98 | 5.39 | 30.61 | 21.00 |
| G9 | CORDNER-15-2 | 1.66 | 8.09 | 6.51 | 26.47 | 20.00 |
| G10 | Alamura (Check) | 1.99 | 15.06 | 9.80 | 22.90 | 55.00 |
| Mean | | 1.94 | 11.78 | 7.96 | 30.85 | 40.00 |
| LSD (0.05) | | 0.78 | 3.34 | 4.32 | 8.67 | 9.00 |
| CV (%) | | 26.30 | 23.40 | 27.00 | 28.80 | 24.10 |

Where, 1-9 scale (1 = immune, 9 =Susceptible, hence 1-3 = resistant, 4 – 6 = medium and 7-9 = susceptible); SPVD=Sweetpotato virus disease, RL=Root length, RG=Root girth, AGFW=above ground fresh weight, HI=Harvest index

**Performance of OFSP genotypes for root dry matter, beta-carotene contents and flowering potential**

The root dry matter contents (DMC) of the evaluated genotypes ranged from 24.0% to 31%, with an overall mean of 27.0% [Table 4]. The highest mean percentage of 31% DMC was recorded for the check variety (Alamura), whereas the lowest mean of 24.0% DMC was recorded for genotype G6 (107031-18-5). The DMC, a key trait in the OFSP variety associated with the mealiness of boiled roots, can impact the wider acceptability of released varieties in areas where this crop is widely consumed, such as East African countries [16]. According to [17] and [18], in order to meet consumer preferences in the humid tropics, an OFSP dry matter level categorized as medium between 24% and 28% and a high dry matter content greater than 28% are required. Accordingly, in this study, three genotypes, such as G3 (13NC9350A-9-3), G7 (105413-5), and the check variety Alamura, had DMCs of 30%, 30%, and 31%, respectively, and can be considered the best genotypes for dry matter content [Table 4].

Another vital nutritional pigment found in the OFSP variety is beta-carotene (BCC), which distinguishes OFSP varieties from white-fleshed ones and is a precursor to vitamin A, one of the most important vitamins for human health. The performance of the evaluated genotypes significantly differed at p<0.05 in test environments [Table 4]. The BCC for genotypes was estimated based on a root flesh color chart following a procedure developed by [38]. As explained by these colour chart standards, the deeper orange root flesh colour of the OFSP genotype indicates a higher beta-carotene concentration. In those standards, it was defined that the ranges for the most favorable genotypes may be defined as orange or intermediate orange, ranging from 5.08 to 8.36 mg/100 g on a fresh weight basis [38]. In this study, check variety (Alamura) had the highest mean value of 7.46 mg/100 g, whereas genotype G9 (CORDNER-15-2) had the lowest mean value of 3.48 mg/100 g. In this study, three genotypes, G1 (MUSG014052-51-5), G3 (13NC9350A-9-3), and G10 (Alamura), showed a desirable BCC range, which is equal to ranges specified as acceptable on a fresh weight basis by [38].

A favorable genotype is one with a relatively high beta-carotene content combined with higher root yields; thus, it is essential to select a suitable genotype for production as a systematic diet in areas where there are nutritional challenges [52]. In terms of flowering ability, most of the evaluated genotypes seldom produced flowers under test environments; however, genotypes with higher flowering potential are desired since they may be used as parents for future hybridization purposes. In this study, genotype G3 performed very well for this trait, i.e., flowing profusely enough that it can be used as a parent for future crossbreeding/hybridization purposes upon further study of the traits of interest [Table 4].

Table 4. Mean dry matter content, beta-carotene content and flowering ability of the evaluated sweetpotato genotypes

| Code | Genotypes | DMC  (%) | BCC*  (gm/100g) | Flowering ability of evaluated genotypes |
| --- | --- | --- | --- | --- |
| G1 | MUSG014052-51-5 | 26.00 | 7.11 | Mostly not flowering or 2% |
| G2 | MUSG014001-3-7 | 25.00 | 3.65 | Mostly not flowering or 3% |
| G3 | 13NC9350A-9-3 | 30.00 | 5.50 | 95% flowering (profusely flowering) |
| G4 | CN1448-49-26-12 | 27.00 | 3.68 | Mostly not flowering or 3% |
| G5 | CN1448-49-28-9 | 27.00 | 3.67 | Mostly not flowering or 5% |
| G6 | 107031-18-5 | 24.00 | 3.72 | Mostly not flowering or 3% |
| G7 | 105413-5 | 30.00 | 3.75 | Mostly not flowering or 5% |
| G8 | 105413-13 | 25.00 | 3.50 | Mostly not flowering or 3% |
| G9 | CORDNER-15-2 | 25.00 | 3.48 | Mostly not flowering or 3% |
| G10 | Alamura (Check) | 31.00 | 7.46 | Mostly not flowering or 10% |
| Mean | | 27.00 | 4.60 |  |
| LSD (0.05) | | 2.00 | 0.55 |  |
| CV (%) | | 15.50 | 7.20 |  |

Where, DMC= Dry matter content, BCC=Beta-carotene content= estimated on a fresh weight basis based on based on colour chart [38].

**Genotype by Environment and Stability Analysis of Genotypes Using GGE-Biplot**

Figure 1 depicts the relative performance of ten OFSP genotypes for root yields across locations based on GGE-biplot. According to the explanations provided by [46], in this study, the first two principal components (PC), PC1 and PC2, were able to explain 84.14% and 10.88% of variation, respectively, with an overall variation of 95.02%, indicating that the first two principal components (PC1 and PC2) adequately explained the variations, which meets the criteria for using a GGE-biplot [Fig 1]. Following an interpretation of their respective values (scores) from a GGE biplot analysis, large positive PC scores for a specific genotype indicate that it is considered a higher average value, whereas those with large negative PC scores indicate that they are considered to be a lower score value [43, 44]. Also, [51] explained that genotypes closer to the origin of the biplot can be considered stable and those farther away as unstable. The authors also explained that genotypes closer to specific environments are more adaptive to that environment.

The performance of a genotype is significantly influenced by the environment in which it is grown [45]. In contrast to widely adapted stable and non-responsive varieties, environment-specific adapted varieties, i.e., unstable and responsive varieties, have a tendency to respond to environmental changes [52]. This affirms the earlier assumption that if all environmental markers fall into one sector, it means that a single cultivar has the highest yield in all environments (across locations and years). In contrast, if environmental markers fall into separate sectors, it suggests that different cultivars excel in different areas.

According to a GGE-biplot analysis, five genotypes, namely G3 (13NC9350A-9-3), G10 (Alamura), G7 (105413-5), G1 (MUSG014052-51-5), and G5 (CN1448-49-28-9), gave the relatively highest root yields, which were above average yields. Whereas G6 (107031-18-5), G8 (105413-13), G9 (CORDNER-15-2), and G4 (CN1448-49-26-12) had the lowest average root yields. Genotypes G5 (CN1448-49-28-9), G2 (MUSG014001-3-7), and G7 (105413-5) had low PC2 values close to zero, indicating that they were relatively stable. Furthermore, [51] explained that stable genotypes are those with a small vector distance from the center of the biplot, as determined by a GGE biplot analysis. Out of the ten genotypes that were tested, G7 (105413-5) is less yielding but more stable than G3 (13NC9350A-9-3) and G10 (Alamura) [Fig. 1]. The vertex genotypes at the polygons G1, G3, G4, G9, and G8 performed either well or poorly, which is in line with previous reports explaining GGE biplot analysis for sweetpotato genotype evaluation [22, 31].

Breeding and evaluating crop varieties for specific areas may be difficult due to the low investment of resources for breeding operations in the variety development process; therefore, selecting a relatively stable genotype with better yield potential across locations is the simplest approach. Genotype G3 performed well almost in all the test environments, i.e., it was found at the apex of the sector, and it is a high yielder with broad adaptation, which can be suggested to be the best genotype for wider production. [51] also explained that the length of the environmental vector shows the magnitude of the influence of genotypic factors, the environments, and their interactions. In this study, Arbaminch locations in both years designated as AM1 and AM2 had relatively large PC1 scores that they better discriminated among evaluated genotypes for root yield. Whereas Hawassa locations designated as HAW1 and HAW2 had PC2 scores close to zero, however, they were considered poor performance environments for root yield during this study period [Fig 1].

The mean yield versus stability view biplot was computed and depicted based on ten OFSP genotypes evaluated in six diverse environments, i.e., three locations vs. two years [Fig 2]. The average environment coordinate (AEC) abscissa (the vertical line that passes through the origin of the biplot) divides genotypes into two groups: high yielders that yield more than the overall mean (right hand side) and lower mean yielders that yield less than the overall mean (left hand side). In this study, genotype G7 was the most stable, with an above-average yield, followed by genotypes G5 and G10. Genotypes G6, G8, and G9 were also the most stable genotypes, but with the least yield performance [Fig 2]. On the other hand, genotype G3 was less stable than all genotypes except G1, which agrees with the earlier report of [29–31]. Although the G3 is less stable than the other genotypes, it is the highest yielder variety in all environments (across locations and years) and has not demonstrated specific adaptability to only one or two environments, reflecting that it can be a suitable genotype in wider production areas.

**GGE-Biplot-based evaluation of test environments for representativeness and discriminating ability**

According to the explanation provided for the GGE-biplot analysis by [41–43, 51], environments with high PC1 scores are considered to be better at distinguishing between genotypes, whereas those with low PC2 values are more representative of the average. The authors also proposed using a biplot to determine the representativeness of a test site, using the average environment as a reference. The average environment coordinate (AEC) abscissa was determined by averaging PC1 and PC2 values across all environments. The proximal angle with the AEC defines the representativeness of an individual location; the smaller the angle between the location vector and the AEC, the more representative the tested site. Based on the GGE explanations, the two environments in this study, designated as HAW1 (Hawassa year 1) and HAW2 (Hawassa year 2), were the most representative in terms of root yields of the tested genotypes, including KOK1 (Koka year 1), whereas KOK2 (Koka year 2), AM1 (Arbaminch year 1), and AM2 (Arbaminch year 2) were the least representative in terms of root yields of the evaluated genotypes [Fig 3]. Two environments designated as AM1 and AM2 are better distinguished between genotypes for root yield, reflecting that they can be better test environments than the others. The current findings of this study are consistent with earlier findings on sweet potato genotype evaluations and recommendations of sweet potato cultivars suitable for varied agro-ecological conditions in Ethiopia [29, 31].

**Conclusion**

The development of orange-fleshed sweetpotato for earliness is highly demanding in areas that are experiencing the adverse effects of climate change, such as erratic rainfall patterns. This group of varieties is believed to be increasingly important because they can reach maturity more quickly, ensuring a harvest even in short growing seasons. Sweetpotato genotypes with the earliness trait can significantly increase production and productivity by ensuring resilience to the climate shocks we are currently facing. Evaluating and selecting the best genotype for that area is of utmost importance. The current study was designed to select suitable genotypes in terms of earliness and high root yields. Accordingly, this study identified genotype G3 (13NC9350A-9-3) as the highest yielder variety in all environments (across locations and years). The GGE biplot also revealed that G3 was the vertex genotype of the polygon that performed the best. Overall, this genotype performed consistently across locations, has a very good level of resistance to SPVD severity, has an acceptable level of beta-carotene and dry matter contents, and matures in a three-month period. Additionally, G3 can be a good fit for relay and intercropping systems due to its semi-erect and non-twining growth habits. Therefore, it can be recommended for verification and release for wider production in the country.

**References**

1. Sheikha AFE, Ray RC. Potential impacts of bioprocessing of sweet potato: Review. Critical Reviews in Food Science and Nutrition 2016; 57:455–71. https://doi.org/10.1080/10408398.2014.960909.
2. Alam M, Rana Z, Islam S. Comparison of the Proximate Composition, Total Carotenoids and Total Polyphenol Content of Nine Orange-Fleshed Sweet Potato Varieties Grown in Bangladesh. Foods 2016; 5:64. https://doi.org/10.3390/foods5030064.
3. Belesova K, Agabiirwe CN, Zou M, Phalkey R, Wilkinson P. Drought exposure as a risk factor for child undernutrition in low- and middle-income countries: A systematic review and assessment of empirical evidence. Environment International 2019a; 131:104973. <https://doi.org/10.1016/j.envint.2019.104973>.
4. FAOSTAT (Food and Agriculture Organization Statistical Data). (2022). Crops and livestock products. Available at [www.fao. org/ faostat] Accessed on 22 May 2024.
5. CSA (Central Statistical Agency). (2022). The Federal Republic of Ethiopia Agricultural sample survey 2021/2022. Report on area and production of major crops (Private Peasant Holdings, Meher Season)**.** Ethiopian Statistical Service, Addis Ababa, Ethiopia
6. CSA (Central Statistics Agency), 2020. The Federal Democratic Republic of Ethiopia

Central Statistical Agency Agricultural Sample Survey 2019/20 Volume I: Report on Area and Production of Major Crops.

7. MoA-FDRE (Ministry of Agriculture of Federal Democratic Republic of Ethiopia. (2024). The National Potato and Sweetpotato Development Strategy: 2024-2030, Addis Ababa, Ethiopia, Ministry of Agriculture of the FDR Ethiopia.
8. Ssali RT, Sseruwu G, Yada B, Ssemakula G, Wasonga C, Grüneberg WJ, et al. Efficiency of the Polycross and Controlled Hybridization Methods in Sweetpotato Breeding in Uganda. Journal of Agricultural Science 2019;11:123. https://doi.org/10.5539/jas.v11n17p123.
9. Jiang W, Li X, Wang R, Du Y, Zhou W. Cross-country health inequalities of four common nutritional deficiencies among children, 1990 to 2019: data from the Global Burden of Disease Study 2019. BMC Public Health 2024;24. https://doi.org/10.1186/s12889-024-17942-y.
10. Laurie SM, Bairu MW, Laurie RN. Analysis of the Nutritional Composition and Drought Tolerance Traits of Sweet Potato: Selection Criteria for Breeding Lines. Plants 2022;11:1804. https://doi.org/10.3390/plants11141804.
11. Belesova K, Agabiirwe CN, Zou M, Phalkey R, Wilkinson P. Drought exposure as a risk factor for child undernutrition in low- and middle-income countries: A systematic review and assessment of empirical evidence. Environment International 2019b;131:104973. https://doi.org/10.1016/j.envint.2019.104973.
12. Pérez-Pazos JV, Rosero A, Martínez R, Pérez J, Morelo J, Araujo H, et al. Influence of morpho-physiological traits on root yield in sweet potato (Ipomoea batatas Lam.) genotypes and its adaptation in a sub-humid environment. Scientia Horticulturae 2021;275:109703. https://doi.org/10.1016/j.scienta.2020.109703.
13. Ritchie H, Roser M. Micronutrient Deficiency. Our World in Data 2024. https://ourworldindata.org/micronutrient-deficiency.
14. Staff Guidance Note on Macroprudential Policy - Detailed Guidance on Instruments. IMF Policy Paper 2014;14. https://doi.org/10.5089/9781498342605.007.
15. Gurmu F, Hussein S, Laing M. The Potential of Orange-Fleshed Sweet Potato to Prevent Vitamin A Deficiency in Africa. International Journal for Vitamin and Nutrition Research 2014;84:65–78. https://doi.org/10.1024/0300-9831/a000194.
16. Gurmu F, Mekonen S. Evaluation of root yield performance of newly bred orange-fleshed sweet potato genotypes in Ethiopia. Journal of Agricultural and Crop Science 2019;7:9–17. https://doi.org/10.33495/jacr_v7i1.18.154.
17. Makunde GS, Andrade MI, Ricardo J, Alvaro A, Menomussanga J, Gruneberg W. Adaptation to mid-season drought in a sweetpotato (Ipomoea batatas [L.] Lam) germplasm collection grown in Mozambique. Open Agriculture 2017;2:133–8. https://doi.org/10.1515/opag-2017-0012.
18. Fuglie KO. Priorities for Sweetpotato Research in Developing Countries: Results of a Survey. HortScience 2007;42:1200–6. https://doi.org/10.21273/hortsci.42.5.1200.
19. Ndunguru J, Kapinga R, Sseruwagi P, Sayi B, Mwanga R, Tumwegamire S, et al. Assessing the sweetpotato virus disease and its associated vectors in northwestern Tanzania and central Uganda. African Journal of Agricultural Research 2009;4:334–43. https://doi.org/10.5897/ajar.9000069.
20. Ngailo S, Shimelis H, Sibiya J, Mtunda K, Mashilo J. Genotype-by-environment interaction of newly-developed sweet potato genotypes for storage root yield, yield-related traits and resistance to sweet potato virus disease. Heliyon 2019;5:e01448. https://doi.org/10.1016/j.heliyon.2019.e01448.
21. Abebe SM, Balcha FG, Mengesha GG. Reaction of introduced orange fleshed sweetpotato (Ipomoea batatas L.) genotypes for virus disease resistance and storage root yield performance under field conditions in Dilla and Hawassa, southern Ethiopia. Indian Phytopathology/Indian Phytopathology 2023;76:243–59. https://doi.org/10.1007/s42360-022-00590-w.
22. Gibson RW, Mpembe I, Alicai T, Carey EE, Mwanga ROM, Seal SE, et al. Symptoms, aetiology and serological analysis of sweet potato virus disease in Uganda. Plant Pathology 1998;47:95–102. https://doi.org/10.1046/j.1365-3059.1998.00196.x.
23. Mekonen S, Tadese T,Gurmu F, Berhanu B. Evaluation of exotic and locally adapted sweetpotato cultivars to major viruses in Ethiopia. Greener Journal of Agricultural Science 2016:069–78. https://doi.org/10.15580/gjas.2016.2.011516011.
24. Valverde RA, Clark CA, Valkonen JPT. Viruses and Virus Disease Complexes of Sweetpotato 2007;1:116–26.
25. Fraile A, Pagan I, Anastasio G, Saez E, Garcia-Arenal F. Rapid Genetic Diversification and High Fitness Penalties Associated with Pathogenicity Evolution in a Plant Virus. Molecular Biology and Evolution 2010;28:1425–37. https://doi.org/10.1093/molbev/msq327.
26. Maquia I, Muocha I, Naico A, Martins N, Gouveia M, Andrade I, et al. Molecular, morphological and agronomic characterization of the sweet potato (Ipomoea batatas L.) germplasm collection from Mozambique: Genotype selection for drought prone regions. South African Journal of Botany 2013;88:142–51. https://doi.org/10.1016/j.sajb.2013.07.008.
27. Gurmu F. Sweetpotato Research and Development in Ethiopia: A Comprehensive Review. Journal of Agricultural and Crop Science 2019;7:106–18. https://doi.org/10.33495/jacr_v7i7.19.127.
28. Andrade MI, Naico A, Ricardo J, Eyzaguirre R, Makunde GS, Ortiz R, et al. Genotype × environment interaction and selection for drought adaptation in sweetpotato (Ipomoea batatas [L.] Lam.) in Mozambique. Euphytica 2016;209:261–80. https://doi.org/10.1007/s10681-016-1684-4.
29. Gurmu F. Stability Analysis of Fresh Root Yield of Sweetpotato in Southern Ethiopia using GGE Bi-Plot 2017;1:1–9. https://doi.org/10.20448/813.11.1.9.
30. Ebem EC, Afuape SO, Chukwu SC, Ubi BE. Genotype × Environment Interaction and Stability Analysis for Root Yield in Sweet Potato [Ipomoea batatas (L.) Lam]. Frontiers in Agronomy 2021;3. https://doi.org/10.3389/fagro.2021.665564.
31. Gemechu GE, Mulualem T, Semman N. Genotype by environment interaction effect on some selected traits of orange-fleshed sweet potato (Ipomoea batatas [L].Lam). Heliyon 2022;8:e12395. https://doi.org/10.1016/j.heliyon.2022.e12395.
32. Yan W, Tinker NA. Biplot analysis of multi-environment trial data: Principles and applications. Canadian Journal of Plant Science/Canadian Journal of Plant Science 2006a;86:623–45. https://doi.org/10.4141/p05-169.
33. Pour-Aboughadareh A, Khalili M, Poczai P, Olivoto T. Stability Indices to Deciphering the Genotype-by-Environment Interaction (GEI) Effect: An Applicable Review for Use in Plant Breeding Programs. Plants 2022;11:414. https://doi.org/10.3390/plants11030414.
34. HwARC (Hawassa Agricultural Research Center). (2015). Sweetpotato production and field management in Ethiopia, Production Manual. Hawassa Agricultural Research Center, Hawassa, Ethiopia.
35. Gruneberg WJ, Eyzaguirre R, Diaz F, Boeck B, Espinoza J, Mwanga, ROM, et al. Procedures for the evaluation of sweetpotato trials. Manual. Lima (Peru). International Potato Center (CIP). 2019; ISBN: 978‐92‐9060‐522‐5
36. Tairo F, Mneney E, Kullaya A. Morphological and agronomical characterization of sweetpotato germplasm collection from Tanzania. African Journal of Plant Science 2008; 077–085.
37. Carey EE, Reynoso D. Procedure for evaluation of pathogen-tested sweetpotato clones. In: Huam_an, Z. (Ed.), Sweetpotato Germplasm Management. Training Manual 3. Evaluation and Breeding. Lima (Peru). International Potato Centre (CIP) 1999.
38. Burgos B. R., Carpio C., Sanchez S. Paola P., Eduardo J., Espinoza, Grüneberg W. A colour chart to screen for high β-carotene in OFSP breeding. The 15th Triennial Symposium of the International Society for Tropical Root Crops (ISTRC), Lima. 2-6 November, ISTRC, Lima, Peru 2009; 47-52.
39. SAS Institute. SAS/STAT user’s guide, version 9.3. Cary. SAS Institute Inc. 2014.vNew York, USA.
40. Gomez KA, Gomez AA. Statistical procedures for agricultural research, 2^nd^ edn, John Wiley and Sons, Inc. New York, Chester, Brisbane, Toronto 1984; 740.ISBN:978-0-471-87092-0.
41. Yan W, Hunt LA, Sheng Q, Szlavnics Z. Cultivar Evaluation and Mega-Environment Investigation Based on the GGE Biplot. Crop Science 2000; 40:597–605. https://doi.org/10.2135/cropsci2000.403597x.
42. Yan W. GGEbiplot—A Windows Application for Graphical Analysis of Multi-environment Trial Data and Other Types of Two‐Way Data. Agronomy Journal 2001; 93:1111–8. https://doi.org/10.2134/agronj2001.9351111x.
43. Yan W, Tinker NA. Biplot analysis of multi-environment trial data: Principles and applications. Canadian Journal of Plant Science/Canadian Journal of Plant Science 2006b; 86:623–45. https://doi.org/10.4141/p05-169.
44. Egea-Gilabert C, Pagnotta MA, Tripodi P. Genotype × Environment Interactions in Crop Breeding. Agronomy 2021; 11:1644. https://doi.org/10.3390/agronomy11081644.
45. Farshadfar E, Sutka J. Biplot analysis of genotype-environment interaction in durum wheat using the AMMI model. Acta Agronomica Hungarica 2006;54:459–67. https://doi.org/10.1556/aagr.54.2006.4.8.
46. Rukundo P, Shimelis H, Laing M, Gahakwa D. Combining Ability, Maternal Effects, and Heritability of Drought Tolerance, Yield and Yield Components in Sweetpotato. Frontiers in Plant Science 2017;7. https://doi.org/10.3389/fpls.2016.01981.
47. Gasura E, Mukasa S. Prevalence and implications of sweetpotato recovery from sweet potato virus disease in Uganda. African Crop Science Journal 2011;18. https://doi.org/10.4314/acsj.v18i4.68648.
48. Shumbusha D, Ndirigwe J, Kankundiye L, Musabyemungu A, Gahakwa D, Ndayemeye PS, et al. ‘RW11-17’, ‘RW11-1860’, ‘RW11-2419’, ‘RW11-2560’, ‘RW11-2910’, and ‘RW11-4923’ Sweetpotato. HortScience 2014; 49:1349–52. https://doi.org/10.21273/hortsci.49.10.1349.
49. Solárová J. Gardner, F. P., Pearce, R. B., Mitchell, R. L. (ed.): Physiology of Crop Plants. Biologia Plantarum 1987; 29:21. https://doi.org/10.1007/bf02902309.
50. Rodrigues NR, Barbosa JL, Barbosa MIMJ. Determination of physico‐chemical composition, nutritional facts and technological quality of organic orange and purple‐fleshed sweet potatoes and its flour. International Food Research Journal 2016; 23:2071-2078.
51. Yan W, Kang MS, Ma B, Woods S, Cornelius PL. GGE Biplot vs. AMMI Analysis of Genotype‐by‐Environment Data. Crop Science 2007; 47:643–53. https://doi.org/10.2135/cropsci2006.06.0374.
52. Laurie SM, Booyse M. Employing the GGE SREG model plus Elston index values for multiple trait selection in sweetpotato. Euphytica 2015; 204:433–42. https://doi.org/10.1007/s10681-015-1359-6.

**Figures**

**Fig 1. Showing the “which-won-where” pattern of tested genotypes for total root yield performance across locations over years b*ased o*n *ge*notype plus genotype-by-environment (GGE) biplot. Where, HAW1=Hawassa year 1; HAW2=Hawassa year 2; Kok1 = Koka year 1; Kok2 = Koka year 2; AM 1 = Arba Minch year 1; AM2= Arba Minch year 2**

Fig 2. The mean performance and stability of 10 evaluated orange-fleshed sweetpotato genotypes in six environments

**Fig 3. The vector view of GGE biplot of 10 OFSP genotypes evaluated across locations over years**

**Supporting information**

**S1 Data. Weather information for the testing locations in 2021 and 2022**

**S1 Table. Description of experimental materials used for the study**
