# Supplementary material for: Evaluation of the performance and stability of early maturing orange-fleshed sweetpotato genotypes in selected areas in Ethiopia
Source: PLoS One. 2024 Oct 25;19(10):e0310273. doi: 10.1371/journal.pone.0310273 (PMC11508074; doi:10.1371/journal.pone.0310273)
Supplement: Attachment — Submitted filename: Rebuttal author revised 23082024.doc [file pone.0310273.s010.doc]

**A letter for responding to reviewers comments**

**Date 23/08/2024**

**To: Academic Editor**

**PLOS ONE**

Subject: **Manuscript Ref:** **PONE-D-23-35204R2**

**Dear Dr Jiban Shrestha,**

Thank you for allowing us to resubmit a revised version of our research paper titled "**Evaluation of the performance and stability of early maturing orange-fleshed sweetpotato genotypes in selected areas of Ethiopia**" to the PLOSONE journal. We are grateful to the PloseOne academic editorial team and reviewers for their time and effort in thoroughly reviewing and providing feedback on our article. Many thanks for that. The reviewers provided very valuable and constructive feedbacks on our manuscript, leading to significant improvements. The manuscript was reshaped, including the abstract, introduction, results, discussion, conclusion, cross check ups of references sections, and plosone formatting, to ensure it better reflects the content.

Dr. Fekadu Gurmu, the co-author of the manuscript, and I took the time to thoroughly revised each point in the manuscript based on feedback provided by reviewers and academic editors.

Dr. Fekadu Gurmu (fekadugb@gmail.com), Cell phone: +251911743625, senior plant breeder and geneticist, Ethiopian Institute of Agricultural Research, Addis Ababa, Ethiopia

**Here is a point-by-point response to the reviewers’ comments and concerns.**

**Editor comments**

“Please review your reference list to ensure that it is complete and correct. If you have cited papers that have been retracted, please include the rationale for doing so in the manuscript text, or remove these references and replace them with relevant current references. Any changes to the reference list should be mentioned in the rebuttal letter that accompanies your revised manuscript. If you need to cite a retracted article, indicate the article’s retracted status in the References list and also include a citation and full reference for the retraction notice”.

**Response**: We checked for these issues, and everything is fine. Thank you!

**Additional Editor Comments:**

The authors need to include all suggestions given by reviewers.

**Response**: We agreed, and all the given suggestions and comments were revised and included in the manuscript. Thank you.

1. If the authors have adequately addressed your comments raised in a previous round of review and you feel that this manuscript is now acceptable for publication, you may indicate that here to bypass the “Comments to the Author” section, enter your conflict of interest statement in the “Confidential to Editor” section, and submit your "Accept" recommendation**.**

Reviewer #5: All comments have been addressed

Reviewer #6: All comments have been addressed

**Response**: Thank you

2. Is the manuscript technically sound, and do the data support the conclusions?

The manuscript must describe a technically sound piece of scientific research with data that supports the conclusions. Experiments must have been conducted rigorously, with appropriate controls, replication, and sample sizes. The conclusions must be drawn appropriately based on the data presented.

Reviewer #5: Partly

Reviewer #6: Yes

**Response**: We have tried to draw a scientifically sound conclusion based on the results we obtained. Thank you

3. Has the statistical analysis been performed appropriately and rigorously?

Reviewer #5: Yes

Reviewer #6: Yes

**Response**: Thank you

4. Have the authors made all data underlying the findings in their manuscript fully available?

The [PLOS Data policy](http://c05y1x9s.r.us-east-2.awstrack.me/L0/http:%2F%2Fwww.plosone.org%2Fstatic%2Fpolicies.action%23sharing/1/010f019170d8129d-9e32d743-eecf-4c8a-9b4b-6a1f6a7705ac-000000/bTaE7nuwruhRG70chn2B-OLL13w=173) requires authors to make all data underlying the findings described in their manuscript fully available without restriction, with rare exception (please refer to the Data Availability Statement in the manuscript PDF file). The data should be provided as part of the manuscript or its supporting information, or deposited to a public repository. For example, in addition to summary statistics, the data points behind means, medians and variance measures should be available. If there are restrictions on publicly sharing data—e.g. participant privacy or use of data from a third party—those must be specified.

Reviewer #5: Yes

Reviewer #6: Yes

**Response**: Thank you

5. Is the manuscript presented in an intelligible fashion and written in standard English?

PLOS ONE does not copyedit accepted manuscripts, so the language in submitted articles must be clear, correct, and unambiguous. Any typographical or grammatical errors should be corrected at revision, so please note any specific errors here.

Reviewer #5: Yes

Reviewer #6: Yes

**Response**: Thank you

6. Review Comments to the Author

Please use the space provided to explain your answers to the questions above. You may also include additional comments for the author, including concerns about dual publication, research ethics, or publication ethics. (Please upload your review as an attachment if it exceeds 20,000 characters)

Reviewer #5: 1. Please check the interpretation on the GGE Biplot, Mean vs Stability. Address the comments in the section as indicated in the manuscript.

**Response**: Thank you for pointing this out. We made corrections and included information concerning **mean vs stability** based on GGE bi-plot analysis, the added information is indicated with track changes in the main document.
2. While quoting references in the text, at several places text abruptly ends in brackets indicating reference number for example : determined using the procedures suggested by [36, 37].

**Response:** We have made a lot of amendments to the text of the document concerning “quoting references in the text”. The change is indicated with track changer. Thank you so much

Please check whether it is the standard way of quoting as per the journal.
3. Other minor comments in the manuscript may be addressed

**Response:** agreed to take action. We have improved by correcting issues such typo errors, spellings, tenses and related issues. The changes were indicated at each section of manuscript with track changers

Reviewer #6: I have read all the previous reviewers comments, and same has been attempted by author. The minor corrections are required as suggested in track changes before publication

**Response:** Thank you. We have addressed the related issues one by one throughout the document, and necessary corrections have been made.

With best regards,

Bililign Mekonnen Senior researcher and coordinator of national root crops research program in Ethiopia, Hawassa, Ethiopia
